# Vaccine priming of rare HIV broadly neutralizing antibody precursors in non-human primates

**DOI:** 10.1126/science.adj8321

**Published:** 2024-05-17

**Authors:** Jon M Steichen, Ivy Phung, Eugenia Salcedo, Gabriel Ozorowski, Jordan R. Willis, Sabyasachi Baboo, Alessia Liguori, Christopher A. Cottrell, Jonathan L. Torres, Patrick J. Madden, Krystal M. Ma, Henry J. Sutton, Jeong Hyun Lee, Oleksandr Kalyuzhniy, Joel D. Allen, Oscar L. Rodriguez, Yumiko Adachi, Tina-Marie Mullen, Erik Georgeson, Michael Kubitz, Alison Burns, Shawn Barman, Rohini Mopuri, Amanda Metz, Tasha K. Altheide, Jolene K. Diedrich, Swati Saha, Kaitlyn Shields, Steven E. Schultze, Melissa L. Smith, Torben Schiffner, Dennis R. Burton, Corey T. Watson, Steven E. Bosinger, Max Crispin, John R. Yates, James C. Paulson, Andrew B. Ward, Devin Sok, Shane Crotty, William R. Schief

**Affiliations:** 1 Department of Immunology and Microbiology, The Scripps Research Institute, La Jolla; CA 92037, USA.; 2 IAVI Neutralizing Antibody Center, The Scripps Research Institute; La Jolla, CA 92037, USA.; 3 Center for HIV/AIDS Vaccine Immunology and Immunogen Discovery, The Scripps Research Institute; La Jolla, CA 92037, USA.; 4 Center for Vaccine Innovation, La Jolla Institute for Immunology; La Jolla, CA 92037, USA.; 5 Department of Integrative Structural and Computational Biology, The Scripps Research Institute; La Jolla, CA 92037, USA.; 6 Department of Molecular Medicine, The Scripps Research Institute, La Jolla, CA 92037, USA.; 7 School of Biological Sciences, University of Southampton, Southampton SO17 1BJ, UK.; 8 Department of Biochemistry and Molecular Genetics, University of Louisville School of Medicine, Louisville, KY 40202, USA.; 9 Division of Microbiology and Immunology, Emory National Primate Research Center; Department of Pathology & Laboratory Medicine, Emory School of Medicine, Emory University, Atlanta, GA, 30322, USA.; 10 Ragon Institute of MGH, MIT & Harvard, Cambridge, MA 02139, USA.; 11 Division of Infectious Diseases and Global Public Health, Department of Medicine, University of California San Diego; La Jolla, CA 92037, USA.; 12 Moderna, Inc., Cambridge, MA 02139, USA.

## Abstract

Germline-targeting immunogens hold promise for initiating the induction of broadly neutralizing antibodies (bnAbs) to human immunodeficiency virus (HIV) and other pathogens. However, antibody-antigen recognition is typically dominated by heavy chain complementarity determining region 3 (HCDR3) interactions, and vaccine priming of HCDR3-dominant bnAbs by germline-targeting immunogens has not been demonstrated in humans or outbred animals. Here, immunization with N332-GT5, an HIV envelope trimer designed to target precursors of the HCDR3-dominant bnAb BG18, primed bnAb-precursor B cells in 8 of 8 rhesus macaques to substantial frequencies and with diverse lineages, in germinal center and memory B cells. We confirmed bnAb-mimicking, HCDR3-dominant, trimer-binding interactions with cryo-electron microscopy. The results demonstrate proof of principle for HCDR3-dominant bnAb-precursor priming in outbred animals and suggest that N332-GT5 has promise to induce similar responses in humans.

## One-Sentence Summary

Proof of principle for generalized germline-targeting vaccine priming in primates.

Humanity could benefit substantially from the design and development of vaccines that induce broadly neutralizing antibodies (bnAbs) to protect against major human pathogens (1). A leading strategy for developing such vaccines is germline-targeting vaccine design (2-7). This strategy aims to induce bnAbs by first priming rare bnAb-precursor B cells and then guiding B cell affinity maturation with a series of rationally designed boosting immunogens (8-11). Clinical proof of principle for germline-targeting vaccine priming was recently demonstrated in the IAVI G001 Phase 1 trial, in which the priming immunogen eOD-GT8 60mer was found to induce responses from diverse VRC01-class bnAb-precursor B cells in 97% of vaccine recipients and to generate substantial frequencies of bnAb-precursor-derived germinal center (GC) and memory IgG B cells (12). However, that trial tested a special case of antibody-antigen interaction in which the antibody HCDR3 plays a minor role. Most antibodies, including most HIV bnAbs, interact with antigen in an HCDR3-dominant manner. Hence, if the germline-targeting strategy is to be employed for induction of other bnAbs to HIV or other pathogens, it must work with HCDR3-dominant antibodies. This requires designing a priming immunogen that can engage a broad pool of bnAb precursors sharing key HCDR3 features but otherwise containing diverse sequences. In the case of HIV, vaccines will need to induce several different classes of bnAbs targeting different epitopes in order to achieve optimal neutralization coverage, increasing the need for effective HCDR3-dominant germline-targeting.

We previously described a generalized method for the design of HCDR3-dominant germline-targeting priming immunogens, and we illustrated the design and testing of HIV envelope (Env) trimer-based N332-GT priming immunogens for the HIV V3-glycan/N332 supersite bnAb BG18 (6). These immunogens induced responses from rare (~1 in 150,000) bnAb-precursor B cells in a BG18 inferred-germline heavy chain knockin mouse model and bound two types of potential bnAb-precursor human naive B cells that share key features with BG18 in ex vivo screens (types I and II). However, the BG18 type I precursors, which show greater HCDR3 similarity to BG18 than type II precursors, occurred at a very low frequency in humans, approximately 1 in 50 million human naive B cells. This frequency was too low to be tested in mouse models and more than 150-fold lower than the VRC01-class precursor frequency in humans (1 in 300,000) for which consistent bnAb-precursor priming was observed in the IAVI G001 trial. Furthermore, consistent priming in humans will likely require activation of BG18 precursors with diverse HCDR3s, heavy chain V genes and light chains, whereas the mouse experiments validated priming of precursors with a single BG18 inferred-germline heavy chain bearing exact HCDR3 junctions from the bnAb itself. The very low BG18 type I human precursor frequency and the need to prime diverse BG18 precursors led us to question the plausibility of consistent priming of BG18 precursors in humans. Reasoning that similar challenges will confront most or all other attempts at HCDR3-dominant bnAb-precursor priming in humans, and that these challenges would be reproduced in non-human primates, we sought to test the concept of HCDR3-dominant germline-targeting in rhesus macaques (RMs). Hence, we evaluated the capacity of the N332-GT5 trimer to prime BG18 type I responses in RMs.

## Rhesus macaque model for BG18 precursor priming

**Fig. 1.**
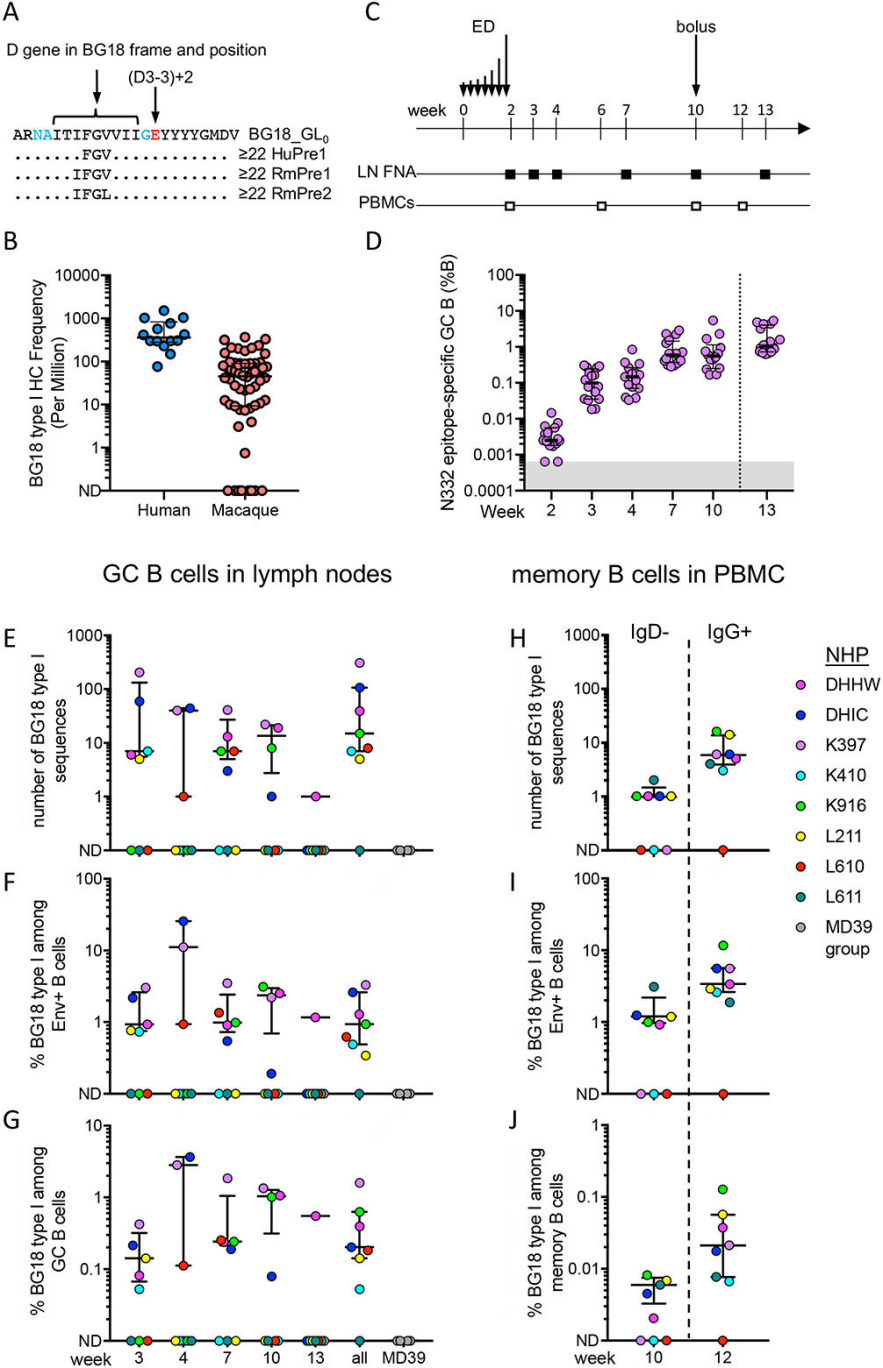
Detection of BG18-like responses in rhesus macaques. (A) The definition of a BG18 type I HC precursor that was used for searching naïve B cells plotted in (B) for humans (HuPre1) and macaques (RmPre1 and RmPre2), where pre1 and pre2 refer to different alleles. The position of the FG(V/L) was allowed to start at position 7, 8, or 9 of the HCDR3. The alignment shows the FG(V/L) starting at position 8. The BG18-inferred germline (BG18_GL_0_) HCDR3 sequence is shown for comparison. Black indicates templated regions of the HCDR3; blue and red indicate junction regions. (B) Frequency of BG18 type I HC sequences in 14 human donors and 60 rhesus macaques. (C) Immunization schedule of NHPs (n=8) with N332-GT5 + SMNP and sampling time points for which sorting and BCR sequencing were carried out. ED, escalating dose; LN FNA, lymph node fine needle aspirate; PBMC, peripheral blood mononuclear cells. (D) Frequency of Env^+^ GC B cells as a percentage of total CD20^+^ B cells. The gray area is set from 0.001% to the median frequency of Env^+^ GC B cells observed in the pre-immunization samples. Lines indicate the median +/− interquartile range. The dotted vertical line indicates that a bolus boost occurred at week 10. (E and H) Number of BG18 type I sequences identified in (E) GC or (H) memory B cells at different time points, with each circle representing a different animal. (F and I) Frequency of BG18-like sequences among Env^+^ B cells from (F) GC or (I) memory B cells. (G and J) Frequency of BG18-like sequences among (G) GC or (J) memory B cells. (E – J) Bars indicate the median +/− interquartile range for the responders. For (E and F) "All" indicates the result of combining all sequences from weeks 3 to 13. In (G) "All" indicates the median frequency among the responders from weeks 3 to 13 at the time points for which responses were detected. In E-G, MD39 group (n=4) indicates Env+ sequences isolated from GC B cells at weeks 3, 4, 7 and 10 after priming with BG505 SOSIP MD39 trimer. ND, not detected.

Although N332-GT5 was designed to target BG18 precursors recombined from human genes, we hypothesized that similar responses might be produced by RMs owing to the relative similarity of their immunoglobulin genes to those of humans. We previously defined BG18 type I human precursors as antibodies with HCDR3s of the same length as BG18 [23 amino acids (aa)], the same heavy chain D gene (D3-3) in the same reading frame and position within the HCDR3, and the same heavy chain joining gene (J_H_6) (Fig. 1A). The definition allows for HCDR3s with diverse V-D and D-J junctions, but our precursor binding studies have shown that there are strong amino acid preferences at some junction positions, including Leu or Phe at the last position of the D gene (which could arise from D-J recombination or somatic hypermutation [SHM]) and a Glu positioned two amino acids past the D gene in the D-J junction [referred to here as (D3-3)+2] (Fig. 1A and table S1) (6). In RMs we used the following criteria for defining BG18 type I precursors: HCDR3 length ≥ 22 aa with a D3-41 gene (homologous to human D3-3) in the same reading frame and in a similar position as D3-3 in BG18 (allowing a shift of ± 1 aa). We note that nearly all bnAbs specific for the N332-supersite have HCDR3 lengths ≥20 aa. We did not use the J_H_ gene as part of the criteria for defining a macaque BG18, because the macaque J_H_6 gene is shorter than human J_H_6 and therefore does not provide an obvious advantage over other J_H_ genes.

In order to determine whether RMs had a BG18 precursor repertoire that was similar to humans, we used the above precursor definitions to compare BG18 precursor frequencies in next-generation sequencing data for 1.1×10^9^ heavy chains from 14 humans (6) and 3.6×10^7^ IgM^+^ heavy chains from 60 RMs (median of 525,916 sequences per RM). We found BG18 type I precursors in 52 out of 60 macaques analyzed, with a median of 45 precursors per million naive B cells, an 8-fold lower frequency than observed for humans (361 precursors per million) (Fig 1B). We evaluated multiple antibody gene features to explain the precursor frequency difference between RM and humans (fig. S1). HCDR3 lengths ≥ 22 aa are five-times more frequent in humans compared to macaques, which can account for the majority of the observed precursor frequency differences between the species (Fig. 1B and fig. S1). We note that these frequencies are substantially higher than the frequencies of N332-GT5-binding precursors (approximately 1 in 50 million in humans), because they do not account for HCDR3 junction determinants or light chain features that affect binding to N332-GT5. Taken together, the data indicate RMs are likely a more stringent model for N332-GT priming than humans due to a lower BG18-like B cell precursor frequency.

## Immunization and serology

We immunized eight Indian RMs bilaterally and subcutaneously in the mid-thigh with 100 μg N332-GT5 trimer and SMNP adjuvant (13) using a slow-delivery escalating-dose (ED) protocol (14-16) followed by a bolus boost at week 10 (Fig. 1C). Draining inguinal lymph node cells were acquired by fine-needle aspiration (FNA) at weeks 2, 3, 4, 7, 10 and 13, and peripheral blood mononuclear cells (PBMCs) and plasma were taken at weeks −2, 2, 6, 10 and 12 (Fig. 1C and fig. S2A). ELISA analyses indicated there was a strong serum-antibody binding response to the BG18 epitope, amounting to approximately 50% of the trimer-specific response at week 10 (fig. S2B-D). We also detected responses to the trimer base and the V3 loop (fig. S2E-H).

## B cell response analysis

The main objective of the study was to determine if the vaccine could induce class-switched B cells with BG18 type I B cell receptors (BCRs). To assess BG18 precursor priming, we employed two different workflows. In the first, N332-GT5 trimer-specific (N332-GT5^+^/N332-GT5^+^ labeled with different fluorophores, “N332-GT5^++”^ hereafter) and N332 epitope-specific (N332-GT5^++^/N332-GT5-KO^−^) GC B cells were sorted from FNA samples, and BCR sequences were recovered by single cell RNA sequencing (figs. S3 and S4). Class switched IgD^−^ memory B cells (MBCs) were also sorted and sequenced from week 10 PBMC samples using this workflow (figs. S3 and S4). In a second workflow, IgG MBCs from week 12 (two weeks post boost) PBMCs that were specific for the N332 epitope (N332-GT5^++^/N332-GT5-KO^−^) were single-cell sorted into 96-well plates and cultured for three weeks, and then BCR sequences were obtained by reverse transcriptase-polymerase chain reaction (RT-PCR) and DNA sequencing for wells that were confirmed N332-GT5-positive by supernatant ELISA (figs. S4 and S5).

Analysis of lymph node cells indicated that all animals produced a GC B cell response specific for the N332 epitope, with frequencies of epitope-specific GC B cells among total B cells increasing ~1600-fold by week 13 compared to pre-immunization (Fig. 1D and fig. S3). The percentage of epitope-specific cells among N332-GT5^++^ GC B cells was high early after immunization (median of 62% at week 2), decreased to 23% at week 10, and then increased to 33% three weeks post boost (fig. S3C). Frequencies of N332-GT5^++^ and N332 epitope-specific GC B cells increased over time after priming series and also increased after the week 10 boost (Fig. 1D and fig. S3, D to G). All animals also generated memory B cells in PBMCs specific for N332-GT5 and the N332 epitope (fig. S4). At week 10 post prime, 0.28% of IgD^−^ memory B cells were N332-GT5^++^, and two weeks post boost (week 12), 1% of IgG^+^ memory B cells were N332-GT5^++^ (fig. S4E). In contrast to GC B cells, the fraction of N332-GT5^++^ B cells that were epitope-specific in PBMCs remained high (68% and 81% for weeks 10 and 12, respectively) (fig. S4G). Overall, the data indicated that the vaccine induced robust antigen-specific and epitope-specific GC and memory B cell responses.

## Detection of BG18 type I responses

From N332-GT5 trimer-binding B cells, we obtained a total of 23,467 heavy chain (HC) and 22,352 heavy chain-light chain paired BCR sequences from GCs, and 1,511 HC with 869 paired BCR sequences from memory B cells. We determined the number and frequency of BG18 type I sequences among GC B cells at each time point and among IgD^−^ memory B cells 10 weeks post prime or IgG^+^ memory B cells two weeks post boost (Fig. 1E-J). As a control, we analyzed 9,339 GC B cell BCR sequences from a separate group of animals immunized contemporaneously using the same regimen with the native-like trimer BG505 SOSIP MD39 that is based on the same isolate, BG505, as N332-GT5 but lacks germline-targeting mutations (5, 16). There were no BG18-like sequences identified in the control group (Fig. 1E-G). In contrast, we detected BG18 type I sequences in trimer-specific GC B cells in 7 of 8 animals at a median frequency among responders of 0.93% when all time points were combined (Fig. 1F). We also detected BG18 type I responses in PBMCs from 7 of 8 animals, with the one PBMC non-responder being positive in the GC B cell samples, meaning that overall, we detected BG18 type I responses in all 8 animals. In PBMCs, the median BG18 type I frequency among trimer-specific B cells from responders was 1.2% in IgD^−^ MBCs at 10 weeks post prime, and 3.4% in IgG^+^ MBCs two weeks post boost (Fig. 1I). Among all IgG^+^ MBCs, the BG18 type I frequency was 0.021% two weeks after the boost, which was 3.6-fold higher than the frequency among IgD− MBCs of responders at the time the boost was given at week 10 (0.006%) (Fig. 1J). By comparison, in the IAVI G001 trial two weeks post boost, VRC01-class IgG^+^ memory B cells were detected at frequencies of 0.088% and 0.13% for low dose and high dose groups, respectively, within an order of magnitude of the BG18-like responses here. We conclude that N332-GT5 consistently induced BG18-like type I B cell responses in RMs.

## Polyclonality of BG18 type I responses

**Fig. 2.**
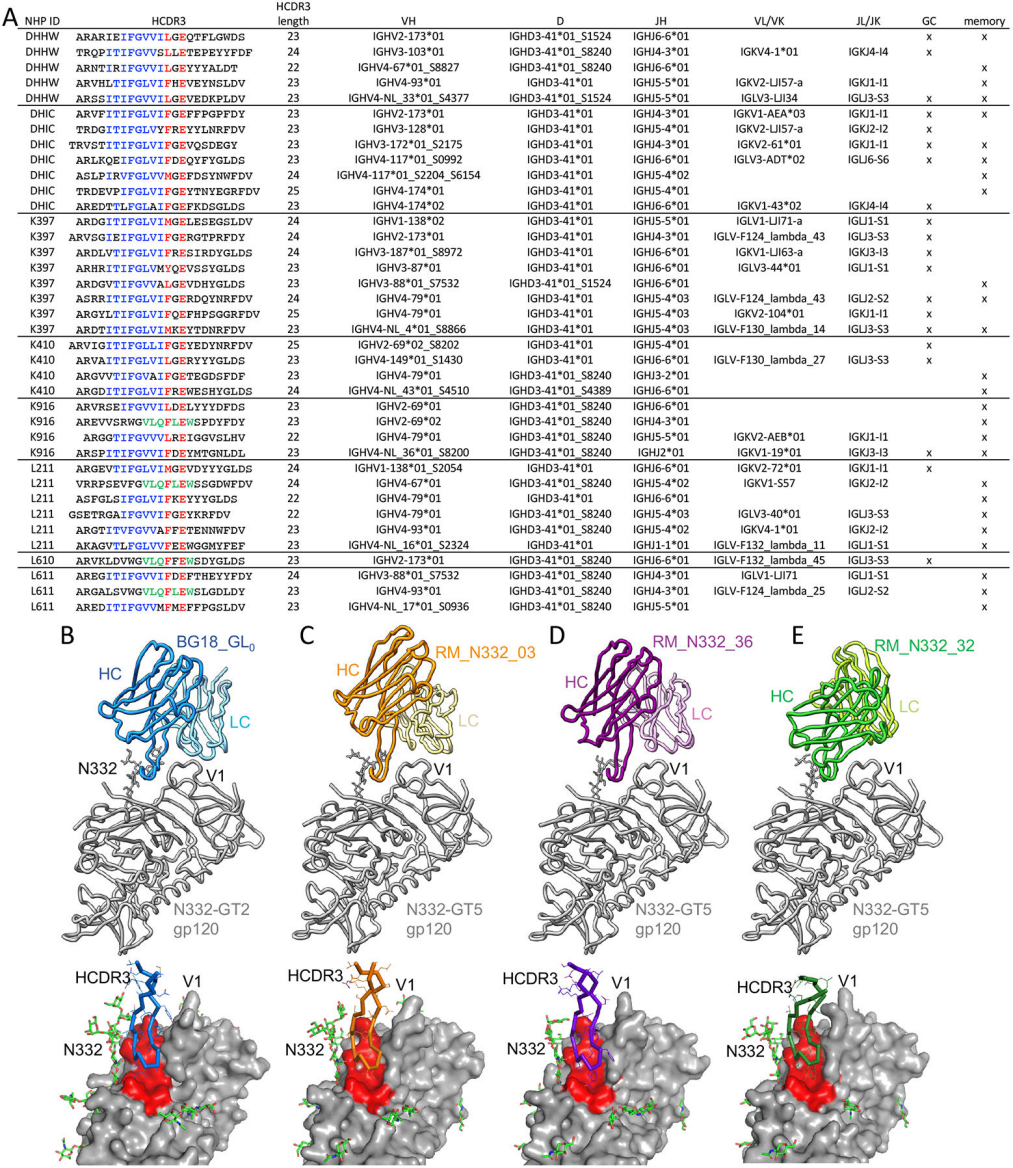
BG18 type I lineages and structural analysis. (A) Gene segment assignment and representative HCDR3 sequence of 38 BG18 type I lineages. The D3-41 gene is colored blue and two critical contact residues are colored red. D3-41 in alternate reading frame is colored green. (B)-(E) upper panels show cryo-EM models of (B) BG18_GL0 Fab in complex with N332-GT2 (PDB ID: 6DFH), (C) canonical BG18 type I macaque Fab RM_N332_03 in complex with N332-GT5 (K_D_, 16 pM), (D) BG18 type I Fab RM_N332_36 with D3-41 in alternate reading frame in complex with N332-GT5 (K_D_, 38 pM), (E) BG18 type I Fab RM_N332_32 with kappa light chain in complex with N332-GT5 (K_D_, 81 pM). (B – E) lower panels show magnified view of HCDR3 interacting with gp120 for each structure; gp120 is colored gray and the N332 epitope (N332-GT2 residues Gly324, Asp325, Val326, Arg327, Met328, Ala329, His330, Ile415, Leu416 and Pro417 or the equivalent position in N332-GT5) colored red; HCDR3s are colored blue, orange, purple, green for BG18_GL_0_, RM_N332_03, RM_N332_36, and RM_N332-32, respectively.

A fundamental hypothesis of germline targeting is that in order to consistently prime rare bnAb precursors in all vaccine recipients, a genetically diverse pool of bnAb precursors must be targeted. The N332-GT5 molecule was designed to engage antibodies containing a specific HCDR3 motif (derived from BG18) but bearing diverse VH and VL gene segments (6). To determine if that germline-targeting goal was met, we carried out a lineage analysis of the BG18 type I antibodies. The sequences were clustered by animal ID, HCDR3 length, and VH gene usage. When two sequences were in separate clusters based on having different but closely related VH gene calls, and the JH gene and non-templated HCDR3 junctions were identical or substantially similar, the sequences were collapsed into the same cluster. This analysis of all time points indicated that N332-GT5 initiated at least 38 unique BG18 type I lineages (Fig. 2A and fig. S6). Eight of the lineages were found in both GC and memory B cells. Across all lineages, HCDR3 lengths ranged from 22 to 25 aa, multiple heavy chain V gene families were used (VH1, VH2, VH3 and VH4), and an approximately equal mixture of lambda and kappa light chains were used. Although the V-D and D-J junctions in the HCDR3s exhibited high sequence diversity, the contact residues were conserved even at non-templated positions including a Glu at the (D3-41)+2 position that was strongly enriched in long but not short HCDR3s (p<0.0001 by two-tailed Fisher's exact test for GC or PBMC data; fig. S7). Furthermore, the last residue of the D gene was replaced with either Leu or Phe in >88% of the lineages, as occurs in the known bnAbs BG18 and PGT121. To inform our sequence analysis, we genotyped the D3-41 gene for each of the eight Indian RMs in the study. The animals were found to have two alleles (*01 and *01_S8240), and all three possible genotypes were represented (*01/*01, *01/*01_S8240, and *01_S8240/*01_S8240). The two alleles each differed from human D3-3 by one amino acid in the BG18 reading frame, and they differed from each other by two amino acids (table S2). BG18 type I lineages were derived from both alleles of D3-41, with *01 representing 24 lineages and *01_S8240 representing 14 lineages. Exemplifying the diversity of BG18-class responses even beyond the strict definition of type I, we found that four of the lineages used D3-41 in an alternate reading frame and position within the HCDR3 but still showed high sequence similarity to the HCDR3 of BG18, including at key contact positions (Fig. 2A). Notably, only the *01_S8240 allele appears to be suitable for making a BG18-like sequence in the alternate reading frame, and the BG18 type I sequences using a D3-41 alternate reading frame were only found in animals with at least one copy of the *01_S8240 allele. We conclude that N332-GT5 induced BG18 type I precursors with diverse HCDR3 junctions and a variety of heavy and light chain V and J genes.

## Structural interactions of BG18 type I Fabs with N332-GT5 trimer

We employed cryo-electron microscopy (cryo-EM) to determine whether N332-GT5-induced BG18 type I BCRs engaged the N332-GT5 trimer with a BG18-like binding mode as predicted by the sequence analysis. We determined structures of three BG18 type I Fabs, each containing distinct genetic features, in complex with the N332-GT5 trimer, and we also determined the structure of the unliganded trimer for comparison (Figs. 2B to E, table S3). The first Fab, RM_N332_03, had a canonical BG18 type I sequence that used the same gene families as BG18 (V_H_4, V_L_3, D3-41). The second, RM_N332_36, also used a V_L_3 light chain (LC) but used D3-41 in a different reading frame and position to produce an HCDR3 closely resembling that of BG18. The third Fab, RM_N332_32, had a BG18 type I HCDR3 sequence but used a kappa chain V gene, V_K_1, the most common kappa chain V-gene family among RM-derived BG18 type I antibodies. Structural analysis confirmed that all three Fabs had a binding mode similar to that of the BG18 inferred germline (BG18_GL_0_), with the HCDR3 engaging the base of V3 and the LC straddling the V1 loop (Figs. 2B-E). The two Fabs that used V_L_3 LCs (RM_N332_03 and RM_N332_36) showed especially high similarity in binding orientation to BG18_GL_0_ in complex with N332-GT2, whereas the RM_N332_32 Fab with a kappa LC showed a somewhat rotated binding orientation relative to BG18_GL_0_, possibly the result of using a kappa LC (fig. S8). The epitope footprint (fig. S9) and HCDR3 conformation (Figs. 2B-E) of all three Fabs showed good similarity to BG18_GL_0_. The fact that RM_N332_36 exhibited such similarity to BG18_GL_0_ demonstrated that D3-41 can form a BG18-like HCDR3 structure in two different reading frames (Figs. 2B-E). BG18 type I antibodies with other D genes and/or reading frames may be relevant to human vaccination as well since we were able to substitute several alternate D gene sequences into the BG18 inferred germline while maintaining high affinity binding to N332-GT5 (table S4). Overall, we conclude that all three BG18 type I Fabs interacted with N332-GT5 using a BG18-like binding mode, which supports our sequence-based definition of a BG18 type I antibody.

## Affinity maturation of BG18 type I and other Env-specific responses

**Fig. 3.**
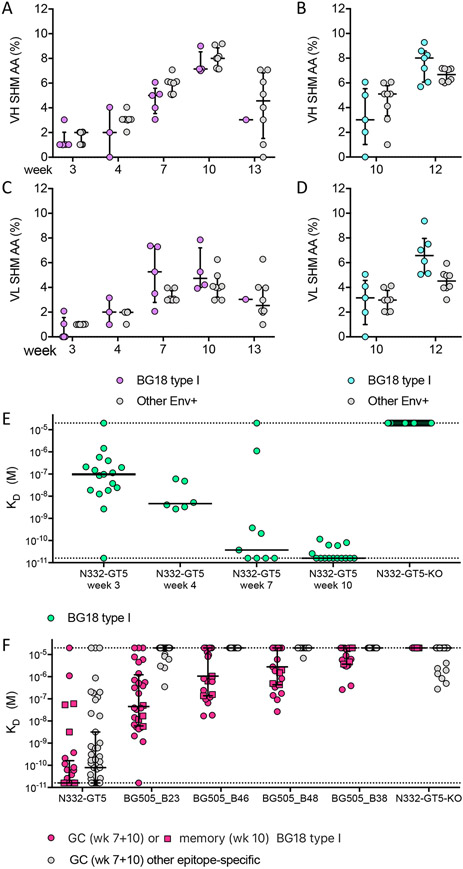
Affinity maturation of BG18 type I and other N332-GT5-elicited antibodies. (A) SHM of VH genes in BG18 type I and other Env^+^ sequences from GC B cells. (B) SHM of VH genes in BG18 type I and other Env^+^ sequences from memory B cells. (C) SHM of VL genes in BG18 type I and other Env^+^ sequences from GC B cells. (D) SHM of VL genes in BG18 type I and other Env^+^ sequences from memory B cells. For (A-D), each dot represents the median SHM for one animal, and lines indicate the median of the medians +/− interquartile range for all animals. (E) SPR K_D_s for BG18 type I Fabs isolated at weeks 3, 4, 7 and 10 post prime from GC B cells binding to N332-GT5 or N332-GT5-KO. The dotted line at 2 x10^−5^ M indicates no binding at the highest concentration tested. The dotted line at 1.6x10^−11^ M represents the approximate upper affinity limit of the instrument. Lines indicate the median with interquartile range. Each data point is representative of 1 to 4 technical replicates. (F) SPR K_D_s for BG18 type I Fabs compared to other epitope-specific Fabs binding to a panel of boost candidates that are more similar to a native trimer than N332-GT5. Data points represent 1 technical replicate. The dotted line at 2 x10^−5^ M indicates no binding at the highest concentration tested. The dotted line at 1.6x10^−11^ M represents the approximate upper affinity limit of the instrument.

Affinity maturation is a requirement for bnAb development, and induction of on-path affinity maturation is thus a key requirement of germline targeting strategies. The BG18 type I BCRs from GCs acquired SHM that increased from a median of 1% V_H_ aa mutation at week 3 to 7% at week 10 (Fig. 3A). BG18 type I BCRs from PBMCs also showed SHM that increased from 3% V_H_ aa mutation at week 10 to 8% at week 12 (two weeks post boost) (Fig. 3B) and there were similar levels of V_L_ mutations in the LC (Figs. 3C-D). BG18 type I antibodies showed similar levels of SHM as other Env^+^ antibodies (Figs. 3A-D).

We produced Fabs for 52 BG18 type I BCRs representing all eight macaques from weeks 3 to 10 post prime; 47 of the Fabs were of GC origin, and five were from memory B cells. None of the BG18 type I Fabs bound to the epitope-knock out protein N332-GT5-KO, demonstrating that all Fabs were epitope specific. Binding affinities of the GC-derived BG18 type I Fabs for N332-GT5 increased over time from a median K_D_ of 98 nM for Fabs isolated at week 3 to <16 pM for Fabs isolated at week 10, representing a >6,000-fold improvement in affinity (Fig. 3E).

In order to determine if the BG18 type I Fabs isolated at later time points (weeks 7 and 10) could bind to trimers that have a more native-like epitope than the priming immunogen, we tested binding to a panel of four trimers based on N332-GT5 but with modified V1 loops. BG505_B23 had both V1 loop N-linked glycosylation sites (N133/N137) reintroduced, but glycan analysis by mass spectrometry revealed that those two glycosylation sites had low glycan occupancy (figs. S11 and S12). Therefore we redesigned the residues surrounding the glycosylation sites and designed three new trimers (BG505_B46, BG505_B48 and BG505_B38) using three different approaches described in the Materials and methods section. All three newly designed trimers showed near complete occupancy of the N137 glycosylation site, and BG505_B46 and BG505_B38 showed near complete occupancy of the N133 glycosylation site (BG505_B48 lacks the N133 site) (fig. S11). BG505_B23, the trimer with low V1 glycan occupancy, showed detectable binding (<20 μM K_D_) to 89% of the BG18 type I Fabs with a median K_D_ of 45 nM. The trimers with high V1 glycan occupancy showed detectable binding to 65%, 57% and 48% of the BG18 type I Fabs for BG505_B46, BG505_B48, and BG505_B38, respectively. Their median K_D_s ranged from 1 μM to >20 μM (Fig. 3F). Thus, 65% of BG18 type I antibodies isolated at weeks 7 and 10 post prime could bind to at least one trimer containing all glycans in the N332 epitope, a necessary requirement of an N332-dependent bnAb. Additionally, BG18 type I antibodies showed the ability to neutralize pseudoviruses expressing BG505-based envelope proteins with N332-GT5 mutations introduced only to the V1 loop (fig. S13). The capacity to bind fully glycosylated trimers and to neutralize a pseudovirus having 12 of the germline-targeting mutations reverted to the wild type residues both indicated that the N332-GT5-elicited Abs are on a pathway consistent with bnAb development. Further, the binding data suggested potential next steps for a sequential immunization strategy, in which the first-boost trimer immunogen will be more native-like than the priming immunogen and will have high specificity for BG18 type I antibodies.

In order to gauge competition for the N332-GT5 epitope that might complicate the induction of appropriate bnAbs and their precursors, we produced 45 Fabs from the week 7 and 10 epitope-specific BCRs that lacked the characteristic BG18-HCDR3 features. These Fabs were chosen by clustering the epitope-specific non-BG18 type I BCR sequences by similarity using methods from the IAVI G001 analysis (12) and producing one Fab from each cluster. The BCR sequences used diverse V, D, and J genes and had diverse HCDR3 lengths (fig. S14). Like the BG18 type I Fabs, the other epitope-specific Fabs showed high affinity for N332-GT5 (median K_D_ of 79 pM) and greatly reduced affinity for N332-GT5-KO (Fig. 3F). In striking contrast to the BG18 type I Fabs, the other epitope-specific Fabs showed either no binding or greatly reduced binding to the four trimers with V1 loop glycosylation sites restored (Fig. 3F), indicating that they are dependent on the V1 glycan hole. The reduced binding to BG505_B23 (the trimer with low glycan occupancy) further suggested that the other Fabs were dependent on specific amino acids present at V1 loop positions 135, 137, or 139 in N332-GT5, most likely K137 and R139, both of which are solvent exposed in unliganded N332-GT5 (fig. S15). Thus, although N332-GT5 elicited diverse competitors for the BG18 epitope in NHPs, the competitors appear to be highly specific for the V1 loop and therefore present a low risk of cross-reactivity with designed heterologous boost immunogens bearing V1 loop glycans.

## Identification of a broader class of BG18-like antibodies.

**Fig. 4.**
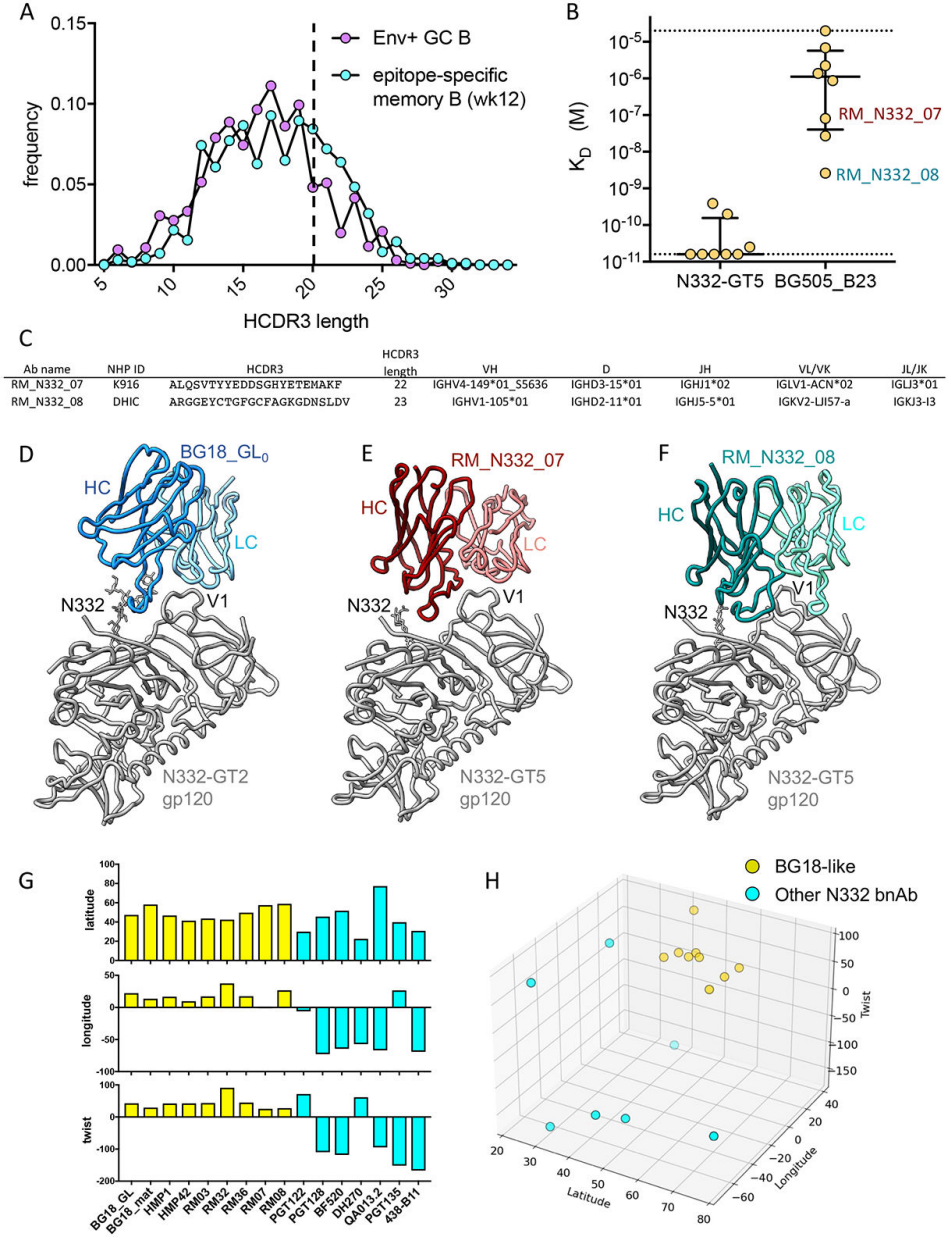
Identification of a broader class of BG18-like antibodies. (A) HCDR3 length distribution of Env^+^ GC B cells and epitope-specific memory B cells isolated at wk 12 after filtering out the BG18 type I sequences. (B) SPR K_D_s of Fabs derived from activated B cell supernatants that were positive for binding to BG505_B23 by ELISA. Each data point represents 1 technical replicate. Lines indicate median +/− interquartile range. The dotted line at 2 x10^−5^ M indicates no binding at the highest concentration tested. The dotted line at 1.6x10^−11^ M represents the approximate upper affinity limit of the instrument. (C) HCDR3 sequence and gene segment assignment of two Fabs that bind with high affinity to the BG505_B23 trimer for which cryo-EM structures were determined. (D) Cryo-EM model of N332-GT2 in complex with BG18_GL_0_ (PDB ID: 6DFH). (E) Cryo-EM model of Fab RM_N332_07 in complex with N332-GT5. (F) Cryo-EM model of Fab RM_N332_08 in complex with N332-GT5. (G) Diagram showing the latitudinal, longitudinal, and HC-LC twist angles for BG18 (PDB ID 6DFG), BG18_iGL0 (6DFH), HMP1 (6NF5), HMP42 (6NFC), RM_N332_03, RM_N332_32, RM_N332_36, RM_N332_07, and RM_N332_08 in yellow bars and seven other N332-dependent bnAbs [PGT122 (4TVP), DH270.6 (6UM6), BF520.1 (6MN7), PGT128 (5ACO), QA013.2 (7N65), PGT135 (4JM2), and 438-B11 (6UTK)] in cyan bars. (H) 3D scatter plot showing latitudinal, longitudinal, and HC-LC twist angles for the antibodies in (G).

After filtering out the BG18 type I sequences from the GC and memory B cell data sets, there remained a large fraction of BCRs containing long (≥ 20 aa) HCDR3s (Fig. 4A), suggesting the potential presence of BG18 type II antibodies, defined as being N332 epitope-specific and having long HCDR3s (≥ 20 aa) and V_L_3 LCs (V_L_3-25, V_L_3-1, or V_L_3-10 in humans) (6). Abs having type II features are inferred to also have a BG18 binding mode. Priming of BG18 type II B cells would be significant as this would indicate that a larger and more diverse pool of BG18-related precursors contribute to an N332-GT5-induced response. Among antibodies isolated from GCs that had a long HCDR3 and a V_L_3 LC, the majority were BG18 type I antibodies (fig. S16), suggesting BG18 type II antibodies may occur at low frequency in macaques. As an alternative approach to searching for antibodies with BG18-like binding properties, we screened the activated supernatants of N332-GT5-sorted week 12 memory B cells against the BG505_B23 trimer (fig. S17), a molecule that is highly selective for binding to BG18 type I antibodies over other epitope-specific antibodies (Fig. 3F). This screen identified BG505_B23-binding supernatants, and sequence analysis revealed these wells to contain antibodies with a wide distribution of HCDR3 lengths. Among the antibodies with HCDR3s ≥ 20 aa, there were both BG18 type I and non-BG18 type I sequences (fig. S17). We produced a subset of Fabs with HCDR3s ≥ 20 aa that were not BG18 type I sequences and found that 7 of 8 showed detectable binding to the BG505_B23 trimer by SPR and 3 of 8 showed high-affinity binding (K_D_<100 nM; Fig. 4B). We determined cryo-EM structures of two of the high-affinity Fabs (RM_N332_07 and RM_N332_08) in complex with N332-GT5 (table S3). RM_N332_07 showed a binding orientation that was highly similar to BG18, with the LC straddling the V1 loop and the HCDR3 making interactions to conserved residues at the base of V3, including R327, H330 and the N332 glycan (Fig. 4D-E and figs. S10A-B). Thus, an antibody having no recognizable BG18 sequence features other than a long HCDR3 can bind to the N332-GT5 trimer in a manner that is highly homologous to BG18. The Fab RM_N332_08 also showed a similar orientation, with the LC straddling the V1 loop and the HCDR3 having partial overlap with the BG18 epitope at the base of V3 but with additional contacts to the V1 loop (Fig. 4F and fig. S10C). We define these antibodies as BG18 type III, having a long HCDR3 (≥ 20 aa) and a binding angle of approach and epitope-footprint similar to BG18, but lacking the HCDR3 sequence features found in BG18 type I sequences and not using V_L_3-25, V_L_3-1, or V_L_3-10 LCs that define a BG18 type II sequence.

We quantified angle of approach for all of the Fab-trimer complex structures obtained in this study, for BG18-class type I and II antibodies isolated from naïve human B cells with N332-GT5 in a previous study (6), and for seven other N332-dependent bnAbs for which structures are available (Fig. 4G-H and fig. S8). The approach angles and relative heavy chain-light chain twist of all seven N332-GT5-reactive Fabs clustered around BG18 and its inferred germline, whereas the other N332-dependent bnAbs approached the epitope with a wide range of angles as has been described previously (17-26). Additionally, all five of the structures of BG18 type I or type III Fabs in complex with N332-GT5 determined in this study showed an epitope footprint that was similar to the BG18 inferred germline (fig. S9). Thus, the BG18 type I and III Fabs structurally characterized here have clear potential to develop into BG18-like bnAbs.

## Conclusions

Developing precision vaccines that induce bnAbs with pre-specified epitopes against HIV and other antigenically diverse pathogens represents an aspirational goal for vaccine design. Achieving that goal will first require design of priming immunogens that consistently recruit bnAb precursors with pre-determined epitope specificities and genetic features conferring potential to mature into bnAbs. Germline-targeting vaccine design provides one strategy to address these challenges, and the IAVI G001 trial provided clinical proof of principle for bnAb precursor induction by a germline-targeting priming immunogen (12). However, the most general form of the priming challenge, which was not addressed by the test in IAVI G001 but will arise in most vaccines, is to design immunogens capable of inducing HCDR3-dominant bnAb precursors. A previous report demonstrated elicitation of V3 glycan-targeting antibodies in RMs (27), but those antibodies did not have pre-defined genetic characteristics and had short HCDR3s (<20 aa), thus they were inconsistent with the known HIV bnAbs that target the V3 glycan epitope. Here, we demonstrated vaccine recruitment of rare HCDR3-dominant bnAb precursors with pre-determined bnAb-associated genetic features including HCDR3 lengths of ≥22aa, in an outbred host with no genetic alterations, thereby validating the proposed generalized germline-targeting vaccine design strategy (6). We further demonstrated outstanding affinity maturation of the bnAb precursors after just a priming immunization.

N332-GT5 was designed to induce diverse human BG18-class type I precursors. In this study, N332-GT5 proved capable of inducing such precursors in RMs with different immunoglobulin genes and rarer precursor frequencies, which altogether suggest that N332-GT5 has substantial potential to prime diverse BG18 type I precursors consistently in humans. Furthermore, we provided evidence that N332-GT5 induced a wider range of non-human primate antibodies with a common BG18-like epitope footprint and binding orientation even in the absence of substantial sequence similarity to BG18, which suggests that N332-GT5 can prime a larger number of BG18-like lineages than originally anticipated. What fraction of BG18-like clones have potential to develop into bnAbs and whether these types of antibodies will be primed in human vaccinations will need to be explored in future studies. Immune responses to N332-GT5 protein adjuvanted with SMNP will be assessed in the phase 1 clinical trial HVTN144 (ClinicalTrials.gov Identifier: NCT06033209).

Having developed a priming immunogen capable of consistently inducing BG18-like bnAb precursors in NHPs, the next challenge will be to develop a series of booster immunogens progressively more similar to the native glycoprotein that can drive maturation to bnAb development. Mouse model experiments testing heterologous first-boost immunogens following N332-GT5 priming demonstrate the feasibility of driving early maturation partway toward bnAb development (28). The information obtained here related to affinities and specificities of both the BG18-like and competitor responses to N332-GT5 priming in NHPs should be helpful to guide the selection of boost immunogens optimized to work in primates. The results of this study represent a major step forward toward development of an HIV vaccine that induces HCDR3-dominant bnAbs and suggest a path to development of precision vaccines to other pathogens.

## Materials and Methods:

### BG18 frequency analysis in naïve B cells from rhesus macaques and humans

#### Consolidated naïve BCR NGS database from 70 Rhesus macaques

Rhesus macaque BCR NGS datasets from 70 animals were downloaded from the NCBI sequence read archive (29-34) or obtained directly from the study authors (35). For datasets contained in Guo et al. (33), preinfection VH1, VH3, and VH4 IgM libraries for each animal were concatenated into single datasets for each animal and processed along with datasets from Vigdorovich et al. (35) using Immcantation (36, 37). To reduce sequencing artifacts, sequences were filtered to include only reads that were observed more than twice. The resulting filtered fastq files served as inputs for the next processing step. All 164 datasets from 70 animals were processed through IgDiscover without germline inference to produce a standardized output for construction of a comprehensive database (29). IGHV expression analysis on the IgDiscover outputs was used to cluster datasets from Zhang et al. (32) and assign animal IDs (zRh1 to zRh15) to each dataset.

#### Sequencing of naïve B cells from four additional rhesus macaques

40x10^6^ frozen PBMC samples from 4 RMs were thawed and recovered in 10% FBS in RPMI. Recovered cells were counted and stained with a B cell staining panel (eBioscience Fixable Viability Dye eFluor 506 (Invitrogen), mouse anti-human CD3 APC-Cy7 (SP34-2, BD Biosciences, mouse anti-human CD14 APC-Cy7 (M5E2, BioLegend), mouse anti-human CD16 APC-eFluor780 (eBioCB16, Thermo Fisher Scientific), mouse anti-human CD20 PerCP-Cy5.5 (2H7, BioLegend), mouse anti-human CD27 PE-Cy7 (O323, BioLegend), goat anti-human IgD FITC (polyclonal, Southern Biotech), mouse anti-human IgG BV786 (G18-145, BD Biosciences), mouse anti-human IgM BV605 (G20-127, BD Biosciences)). Approximately 1.5 million CD20+IgG− B cells were sorted for each animal into RPMI containing 50% FBS using a FACSymphony S6 (BD Biosciences). Immediately after sorting, cells were centrifuged at 500 x g for 10 minutes and resuspended in 350ul of buffer RLT (Qiagen, 79216) by vortexing. Lysed cells were immediately frozen at −20C then shipped to the Emory National Primate Research center for repertoire sequencing. Table S5 lists the commercial antibodies used in this study.

The protocol for RM repertoire sequencing was obtained by courtesy of Dr. Daniel Douek, NIAID/VRC. RNA was isolated using QIAGEN RNeasy kits (Valencia, CA) with an input of 1.5M cells for REt18, RGp18, RPb18, and RPz18. Reverse transcription (RT) was performed using Clontech SMARTer cDNA template switching which involves 5′ CDS oligo(dT) (12 μM) being added to RNA and incubated at 72°C for 3 minutes and 4°C for at least 1 minute. The RT mastermix was made using 5x RT Buffer (250 mM Tris-HCl (pH 8.3), 375 mM KCl, 30 mM MgCl_2_), Dithiothreitol, DTT (20 mM), dNTP Mix (10 mM), RNase Out (40 U/μL), SMARTer II A Oligo (12 μM), Superscript II RT (200 U/μL) and was added to the reaction and incubated at 42°C for 90 minutes and 70°C for 10 minutes. First-strand cDNA was purified using AMPure XP beads (Beckman Coulter). Following RT and purification, two PCR rounds were carried out to generate immunoglobulin amplicon libraries that were compatible with Illumina sequencing. All oligos were ordered from Integrated DNA Technologies. The first PCR amplification was carried out using KAPA Real-Time Library Amplification Kit (Roche Diagnostics). cDNA was combined with master mix containing 2X KAPA HiFi HS RT PCR Master Mix, 12 μM μL 5PIIA and 5 μL RhIgM Constant Primer (2 μM). The amplification was monitored using real-time PCR and was stopped at 19 cycles during the exponential phase. The amplified products were again purified using AMPure XP beads. A second round of PCR amplification was carried out for addition of barcodes and Illumina adaptor sequences. Each sample contained 2X KAPA HiFi HS RT PCR Master Mix 2x, Nuclease-free water, 10 μM of P5_Seq BC_XX 5PIIA oligo, 10 μM of P7_ i7_XX RhIgM oligo and were combined with amplified Immunoglobulin from the first round PCR and amplified for 7 cycles using real-time PCR monitoring. The P5_Seq BC_XX 5PIIA primers contain a randomized stretch of four to eight random nucleotides followed by a barcode sequence and this step was followed by purification with AMPure XP beads. A final PCR step was performed for addition of remaining Illumina adaptors by mixing master mix (2X KAPA PCR Master Mix, 10 μM P5_Graft P5_seq, Nuclease-free water), 10 μM of P7_ i7_XX RhIgM oligo and amplified products from the previous PCR step followed by purification with AMPure XP beads. The quality of the library was assessed using Agilent Bioanalyzer 2100 and quantified on a Qubit 4 Fluorometer with 1X dsDNA HS Assay Kit. The amplicon libraries were pooled and sequenced across two Illumina MiSeq v3 runs as a 309 paired-end to obtain a sequencing depth of 10M reads/sample. Sequencing was conducted at the Emory National Primate Research Center Genomics Core Laboratory (http://www.yerkes.emory.edu/nhp_genomics_core).

List of Oligonucleotides from IDT

CDS Oligo (dT): TTTTTTTTTTTTTTTTTTTTTTTTTVN

SMARTer II A Oligo: AAGCAGTGGTATCAACGCAGAGTACATrGrGrG

5PIIA: AAGCAGTGGTATCAACGCAGAGT

RhIgM_Constant_Discover: GGGGCATTCTCACAGGAGACGAGGGGGAAAAG

P5_Seq BC_XX 5PIIA: CACGACGCTCTTCCGATCT 4-8xN AACCACTA AAGCAGTGGTATCAACGCAGAGT

P7_i7_XX_RhIgM_Discover: CAAGCAGAAGACGGCATACGAGAT CGATCGAA GGGGCATTCTCACAGGAGACGAGGGGGAAAAG

P5_Graft P5_seq: AATGATACGGCGACCACCGAGATCTACACTCTTTCCCTACACGACGCTCTTCCGATCT

#### Annotating BCR sequences from 74 rhesus macaques

Datasets from all 74 animals were then annotated to AIRR format (38) using the AIRR module from the SADIE library v0.5.4 (https://github.com/jwillis0720/sadie) using the “macaque” option and as the input annotation species and the adaptable penalty set to true. Results were filtered for only productive reads and converted to parquet format using snappy compression. Only animals that had >100,000 IgM sequences were included in the precursor frequency analysis (60 animals in total). For querying, we used Spark facilitated by AWS EMR service as described in Steichen et al. (6). Example notebooks including instructions on how to access annotated and raw sequences can be found at our repository, https://github.com/schieflab/steichen2023

#### BG18 precursor frequency estimates in rhesus macaques and humans

We analyzed NGS datasets of 1.1 billion human BCR heavy chain sequences from 14 human donors that were previously described (6, 39, 40), as well as 95.4 million macaque BCRs from 60 macaques, using the Spark analytics engine on the AWS EMR platform (EMR 6.4.0). We utilized the precursor definitions provided in fig. S1E and performed the analysis using PySpark scripts. The scripts can be accessed at https://github.com/SchiefLab/Steichen2023, along with instructions for setting up an EMR cluster. Each node was configured with Spark, JupyterEnterpriseGateway, Hadoop, and JupyterHub via the EMR node configuration interface. We then used the EMR notebook interface to run PySpark scripts and analyze precursor frequencies. A precursor frequency was estimated by taking the number of BCR sequences that met a specific query definition and dividing it by the total number of BCRs for each donor. We then multiplied these numbers by 1,000,000 and plotted them as frequencies per million. Median per species was shown on log scale graphs. All plots were generated using GraphPad Prism.

### Animals and immunizations

Indian rhesus macaques (*Macaca mulatta*) were housed at AlphaGenesis Inc. and maintained in accordance with NIH guidelines. This study was approved by the Alpha Genesis Inc. Institutional Animal Care and Use Committee (IACUC). All animals were between 2-3 years old at the time of the priming immunization. For the N332-GT5 plus SMNP escalating-dose immunization group, 8 RMs (4 females and 4 males) were immunized with 50 μg N332-GT5 and 375 μg SMNP each side. For the MD39 plus SMNP escalating-dose immunization group, 4 RMs (2 females and 2 males) were immunized with 50 μg MD39 and 375 μg SMNP each side. All immunizations were given subcutaneously (s.c.) in the left and right mid-thighs. For priming, a 7-dose 12-day escalating dose strategy was used (15) and a bolus boost immunization was given at week 10. Data from the MD39 group have been previously published (16).

### Genotyping rhesus macaques

To genotype IGHD3-41, we utilized targeted long-read Pacific Biosciences single molecule real-time (SMRT) sequencing data generated for each animal in our study cohort (n=8). Sequencing data was generated by adapting our published human immunoglobulin (IG) loci targeted enrichment protocol (41, 42). Briefly, a custom oligo probe panel was designed (“HyperExplore”, Roche) using IG heavy chain (IGH), kappa (IGK), and lambda (IGL) genomic region sequences from the RM genome reference build (RheMac10) and alternative haplotype assemblies from Cirelli et al. (15) as sequence targets.

High molecular weight genomic DNA was isolated from PBMCs collected from each animal using the DNeasy kit (Qiagen). DNA (1 to 2 μg) was then sheared using g-tubes (Covaris) and size selected using a Blue Pippin instrument (Sage Science). Size-selected DNA was End Repaired and A-tailed using the standard KAPA library protocol (Roche), followed by the ligation of sample-specific sequence barcodes and universal primers. PCR amplification was performed for 8-9 cycles using PrimeSTAR GXL polymerase (Takara), and the resulting products were further size-selected and purified using 0.7X AMPure PB beads (Pacific Biosciences). Target-enrichment hybridization was performed using IGH/K/L-specific oligonucleotide probes (Roche). Target fragments were recovered using streptavidin beads (Life Technologies), followed by a second round of PCR amplification for 16-18 cycles using PrimeSTAR GXL (Takara). Long-read sequencing libraries were prepared using the SMRTbell Express Template Preparation Kit 2.0 (Pacific Biosciences), including Damage Repair and End Repair mix to repair nicked DNA, followed by the addition of an A-tail and overhang ligation with SMRTbell adapters. Libraries were then treated with a nuclease cocktail to remove unligated input material and purified with 0.45X AMPure PB beads (Pacific Biosciences). The resulting libraries were pooled and sequenced on the Sequel IIe system (2.0 chemistry; 30h movies) to generate high fidelity reads, with average read accuracy 99.891325%.

High-fidelity reads for each animal were mapped to the RheMac10 genome reference. To genotype IGHD3-41, phased single nucleotide variants representing two distinct alleles were resolved from HiFi reads spanning the IGHD3-41 gene. At least 10 representative HiFi reads were required to include a given allele in the genotype of an animal.

### Analysis of plasma by ELISA

ELISA plates (Corning^™^ 96-Well Half-Area Plates, Catalog # 3690) were precoated with anti-His antibody (1μg/ml; Genscript) or PGT128 Fab (1μg/ml) on Day1. The V3-peptide (1μg/ml) was directly coated on plates on Day1. Plates were incubated overnight at 4°C. Plates were washed with PBST (PBS + 0.2% tween 20) and HIV trimers were captured for 2h at room temperature on Day 2. Plasma serially diluted in blocking buffer (PBST, 1% (w/v) FBS) was added for 1h at at 37°C and 80% humidity. Plates were washed three times and 25 μl of Peroxidase AffiniPure Donkey Anti-Human IgG (H+L) (Jackson ImmunoResearch Catalog # 709-035-149) was added to each well at 1:5,000 dilution in PBST + 1% FBS. After 1h incubation at RT, plates were washed 3X and TMB Chromogen Solution (Thermo Fisher Catalog # 002023) substrate was added. To stop the reaction, 25 μl 0.5 M H2SO4 were added after 5 minutes. Absorption was read at 450 and 570 nm on a Molecular devices VersaMax plate reader (Versamax, USA). Background subtraction was performed by subtracting the 570 nm value from the corresponding 450 nm value. Area Under Curve (AUC) was calculated in Prism (GraphPad Software, La Jolla, USA) by the trapezoidal method, which is based on connection of a straight line between every set of adjacent points defining the curve, and on a sum of the areas beneath these areas.

### Lymph node fine needle aspiration

Lymph node fine needle aspirates (LN FNAs) were used to sample the left and right draining inguinal LNs (iLNs) which were identified by palpation and were performed by a veterinarian. A 22-gauge needle attached to a 3-mL syringe was passed into the LN up to 5 times. Samples were transferred into RPMI containing 10% fetal bovine serum (FBS) and 1x penicillin/streptomycin (pen/strep). Ammonium-Chloride-Potassium (ACK) lysing buffer was used if the sample was contaminated with red blood cells. LN FNA samples were frozen down and stored in liquid nitrogen until analysis.

### Flow cytometry and sorting

Frozen FNA or PBMC samples were thawed and recovered in 10% FBS in RPMI. The recovered cells were counted and stained with the appropriate staining panel. Fluorescent antigen probes were prepared by mixing fluorophore-conjugated streptavidin (SA) with incremental amounts of either biotinylated N332-GT5 or N332-GT5-KO in 1x PBS at room temperature (RT) over the course of 45 min. The KO probe, N332-GT5-KO, was first added to the cells for 20 minutes, followed by the addition of WT N332-GT5 for 30 minutes, and then with the surface antibodies for 30 minutes at 4°C, similar to previously described protocols (15, 16). For samples being sorted, anti-human TotalSeq-C hashtag antibodies (BioLegend) were added to each individual sample at a concentration of 2 μg per 5 million cells at the time of addition with the antibody master mix. 10% FBS in RPMI (R10) supplemented with 1x pen/strep and 1x GlutaMAX was used as the FACS buffer. Pre-immunization samples were acquired on either a FACSFusion (BD Biosciences) or a FACSymphony S6 (BD Biosciences), while post-immunization samples were sorted on a FACSymphony S6 (BD Biosciences). Depending on the timepoint, some LN FNA samples were sorted for both Env^+^ (N332-GT5-BV421^+^N332-GT5-BV650^+^) and Env^+^KO^−^ (N332-GT5-BV421^+^N332-GT5-BV650^+^/N332-GT5-KO-PE^−^) GC B cells. The indexed V(D)J, Feature Barcode and Gene Expression libraries of sorted LN FNA or PBMC samples were prepared following the protocol for Single Indexed 10X Genomics V(D)J 5' v.1.1, with Feature Barcoding kit (10X Genomics). Custom primers designed to target RM BCR constant regions were used at concentrations previously described (16).

For LN FNA data inclusion in GC B cell gating, a threshold of 250 total B cells in the sample was used. For Env-specific GC B cell gating, a threshold of 75 total GC B cells was used. The limit of detection (LOD) was calculated as the median of [3/(number of B cells recorded)] from the LN FNA samples at the pre-immunization timepoint. Left inguinal LN samples from two animals (DHHW and DHIC) at week 10 were excluded from antigen-specific GC B cell analysis. Week 3 and week 4 samples were gated on all live cells.

The following reagents were used during staining: Alexa Fluor 647 streptavidin (Invitrogen), BV421 streptavidin (BioLegend), BV650 streptavidin (BioLegend), eBioscience Fixable Viability Dye eFluor 506 (Invitrogen), mouse anti-human CD3 BV786, APC-Cy7 (SP34-2, BD Biosciences), mouse anti-human CD4 BV711 (OKT4, BioLegend), mouse anti-human CD8a APC-eFluor780 (RPA-T8, Thermo Fisher Scientific), mouse anti-human CD14 APC-Cy7 (M5E2, BioLegend), mouse anti-human CD16 APC-eFluor780 (eBioCB16, Thermo Fisher Scientific), mouse anti-human CD20 Alexa Fluor 488, PerCP-Cy5.5 (2H7, BioLegend), mouse anti-human CD27 PE-Cy7 (O323, BioLegend), mouse anti-human CD38 APC (OKT10, NHP Reagents), mouse anti-human CD71 PE-CF594 (L01.1, BD Biosciences), mouse anti-human PD-1 BV605 (EH12.2H7, BioLegend), mouse anti-human CXCR5 PE-Cy7 (MU5UBEE, Thermo Fisher Scientific), goat anti-human IgD FITC (polyclonal, Southern Biotech), mouse anti-human IgG Alexa Fluor 700, BV786 (G18-145, BD Biosciences), mouse anti-human IgM PerCP-Cy5.5, BV605 (G20-127, BD Biosciences), TotalSeq-C anti-human Hashtag antibody 1-10 (LNH-94 and 2M2, BioLegend), and TotalSeq-C0953 PE streptavidin (BioLegend).

### 10X BCR sequencing

Cell Ranger v.3.0.2 was used for full-length VDJ read assembly. A custom RM germline VDJ reference was generated using databases published previously (15, 30, 31). The constants.py file in the Cell Ranger python library was modified to increase the maximum CDR3 length to 110 nucleotides. N332-GT5-KO-binding in GC B cells and memory B cells (MBCs) were determined by the PE-hashtag read counts, which were assessed by flow cytometry for each timepoint. Depending on the sorting strategy and the ratio of sorted N332-GT5^+^ and N332-GT5^+^KO^−^ GC B cells in each catch tube, PE-hashtag thresholds were defined per tissue and timepoint based on the flow cytometry analyses. For GC B cells from timepoints of week 3 to 7, a threshold of 100 PE-hashtag read counts was used. A sequence with a read count equal to or less than 100 PE-hashtag read counts was considered as epitope-specific (N332-GT5^+^KO^−^). For week 10 and 13, a threshold of 200 PE-hashtag read counts was used. For MBCs, a threshold of 300 PE-hashtag read count was used. Filtered VDJ contigs from the VDJ pipeline were used in further analysis.

### Longitudinal lineage and somatic hypermutation analysis of BCR sequences

The following analysis was used to generate fig. S6. For lineage analysis and SHM calculations used in Figs. 2-3, the analysis was carried out as described in “Bioinformatic analysis of BCR sequences”. The V(D)J filtered contig output from Cell Ranger was further analyzed using packages from the Immcantation Portal (36, 37). An IgBLAST database was built from the custom RM germline VDJ reference. The Change-O pipeline was used to parse the 10X V(D)J sequence output from Cell Ranger into an AIRR community standardized format, to allow for more downstream analysis using the Immcantation Portal. Clonal lineages were calculated for each animal using DefineClones.py with the appropriate clustering threshold value as determined by the disToNearest command from the SHazaM package. Inferred germline V and J sequences from the RM reference were added with CreateGermline.py. The germline D gene sequences and N nucleotide additions were masked from analysis since these cannot be accurately predicted. The total number of mutations (within V- and J-genes) for each heavy chain (HC) was determined by counting the number of nucleotide changes between the observed sequence and the predicted germline sequence with the observedMutations command within SHazaM. For analysis of total HC mutations, all productive HC contigs were analyzed. Sequences in which the VH call aligned to alleles IGHV3-100*01, IGHV3-100*01_S4205, IGHV3-100*01_S4375, IGHV336*01_S5206, IGHV3-36*01_S6650, IGHV3-NL_11*01_S5714, IGHV4-79-a, IGHV4-NL_1*01_S0419 were found to have a tremendously high number of substituted nucleotides at all timepoints compared to their inferred germline sequences, observed previously (16). This observation was likely due to poor V-gene assignment in an incomplete RM V(D)J reference library and these sequences were excluded from further analysis. Clones with paired HC-LC BCR sequences were used when building clonal trees. Maximum-likelihood lineage trees were built for clonal families using Dowser (43) with the pml method within the GetTrees function. For the lineage trees, the branch length indicates the estimated number of total HC mutations and its most recent common ancestor in lineage.

### Cell Lines

MS40L-low (44) or irradiated 3T3msCD40L feeder cells (45) were used in single B cell culture assays. MS40L-low cells were maintained in Iscove's Modified Dulbecco's medium with GlutaMAX (IMDM) (Gibco), supplemented with 10% heat-inactivated Fetal Bovine Serum (FBS) (Omega Scientific), 100 Units/ml Penicillin, 100 μg/ml Streptomycin (1% Pen-Strep) (Gibco), and 55 μM 2-Mercaptoethanol (2-ME) (Gibco) before sorting. For single B cell sorting, MS40L-low cells were maintained in B cell activation media: RPMI-1640 with GlutaMAX supplemented with 10% FBS, 55 μM 2-ME, 1% Pen-Strep, 10 mM HEPES (Gibco), 1 mM Sodium Pyruvate (Gibco), 1% MEM NEAA (Gibco), while irradiated 3T3msCD40L cells were thawed on day of sorting and maintained in IMDM supplemented with 10% FBS, 1X MycoZap Plus-PR (Lonza). HEK293T cells (ATCC) were used to produce viruses and maintained in complete Dulbecco's modified Eagle's medium (DMEM) (Gibco), supplemented with 10% FBS, 2 mM L-glutamine (Gibco), and 1% Pen-Strep. TZM-bl cells (NIH AIDS Reagents Program) (RRID:CVCL_B478) were maintained in complete DMEM and used as target cells in pseudovirus neutralization assays. All cell lines were maintained at 37°C in a humidified atmosphere of 5% CO_2_.

### Isolation of N332-GT5 epitope-specific memory B cells by flow cytometry for culture

Fluorescently labeled antigens used for sorting were generated on the day of the sort by incubating 4 μM biotinylated N332-GT5 WT with streptavidin Alexa Fluor 647 (N332-GT5-AF647) (Invitrogen, cat# S21374) and streptavidin Alexa Fluor 488 (N332-GT5-AF488) (Invitrogen, cat# S11223) separately; and 4 μM biotinylated N332-GT5 KO with BV421 streptavidin (N332-GT5 KO-BV421) (BD Biosciences, cat# 563259) at a 2:1 molar ratio at room temperature for 1 hour in the dark. Washes, staining master mix, and sample preparation were carried out in cold sterile FACS buffer composed of 2% FBS in DPBS (Gibco). Cryopreserved peripheral blood mononuclear cells (PBMCs) from N332-GT5-immunized rhesus macaques were thawed, washed, and stained with antibody master mix of CD3 (clone SP34-2, BD Biosciences, cat# 557757), CD4 (clone OKT4, Biolegend, cat# 317418), CD8 (clone RPA-T8, BD Biosciences, cat#557760), CD14 (clone M5E2, BD Biosciences, cat# 561384), CD20 (clone 2H7, Biolegend, cat# 302326), IgM (clone MHM-88, Biolegend, cat#314508), IgG (clone G18-145, BD Biosciences, cat# 564230), and N332-GT5 KO-BV421 at 4°C for 15 min in the dark. Next, N332-GT5-AF647 and N332-GT5-AF488 WT antigens were added and incubated for an additional 30 min at 4°C in the dark. All antibodies were added at a 1:100 dilution in 100μL and antigens at 100 nM final. Finally, 1:300 LIVE/DEAD fixable cell dye (Invitrogen, cat# L34957) was added and incubated for 15 min at 4°C in the dark, washed, and resuspended to the desired volume. Double positive N332-GT5 WT, KO negative epitope-specific memory B cells (N332-GT5^++^/N332-GT5-KO^−^ IgM^−^IgG^+^ B cells) were single-cell sorted into 96-well plates pre-seeded with appropriate feeder cells using a BD FACSMelody Cell Sorter. Post-sort analyses were done with FlowJo 10.7.2 (FlowJo, LLC).

### Single memory B cell culture and Activation

Single B cells were cultured and expanded as previously described with some modifications (44, 45). Briefly, MS40L-low feeder cells (MS40L-low cultures) were pre-seeded 24 hours before in 96-well plates at a density of 3 × 10^3^ cells/well in 100 μL B cell activation media and supplemented with 100 μL 2X cytokines on the day of sort: 20 ng/mL IL-4 (Peprotech, cat# 200-04), 20 ng/mL IL-21 (Peprotech, cat# 200-21), 200 ng/mL IL-2 (Peprotech, cat# 200-02), 200 ng/mL BAFF (Peprotech, cat# 310-13). Media was replaced with 100-200 μL B cell activation media with 1X cytokines at day 4, 8, 12, 15, and 18(44). For animals K397 and K916, irradiated 3T3msCD40L (3T3 cultures) feeder cells which can be seeded on the day of sorting and have similar activation efficiency to MS40L-low cells were used to complete sample sorting. Irradiated 3T3msCD40L feeder cells were seeded the day of sorting at a density of 4 × 10^4^ cells/well in IMDM complete medium and supplemented with 50 ng/mL each of IL-4, IL-21, IL-2, 100 ng/mL Anti-rhesus IgG (H+L) (Bio-Rad, cat# AAI42) and cultured for 14 days(45). B cell culture supernatants from days 12, 14, 15, and 18 were harvested and used for IgG and antigen ELISA. After media removal, B cells in 96-well plates were frozen at −80°C without any additional buffer and used for B cell receptor sequence analysis.

### ELISA for B cell activation screening

B cell culture supernatants from days 12, 14, 15, and 18 were used to screen for B cell activation (IgG expression) and antigen binding. Briefly, 96-well half area high binding plates (Corning cat# 3690) were coated overnight at 4°C with AffiniPure F(ab’)_2_ Fragment Goat Antihuman IgG (H+L) (Jackson Immunoresearch, cat# 109-006-088) diluted 1:500 in PBS, or 6x-His tag monoclonal antibody diluted 1:500 in PBS (Invitrogen, cat# MA1-21315). Plates were washed 3x with wash buffer composed of 0.01% or 0.05% Tween-20 in PBS for antigen and IgG ELISA, respectively, and blocked with 3% BSA/PBS for 1 hour at RT. For antigen ELISA, His-tagged N332-GT5 WT, N332-GT5 KO, and B23 were separately added to anti-His coated plates at 1 μg/mL in 1% BSA/PBS and incubated for 1 hour at RT and washed 3x. 25 μL control antibodies (BG18, DEN3) serially diluted in 1% BSA/PBS, and B cell culture supernatants were added onto plates and incubated for 1 hour at RT. Plates were washed 3x, and detected with alkaline phosphatase-conjugated anti-Human IgG Fc fragment-specific antihuman IgG (Jackson Immunoresearch, cat# 109-055-098) diluted 1:1000 in 1% BSA/PBS and incubated for 1 hour at RT. Plates were developed with phosphatase substrate (Sigma-Aldrich, cat# S0942), and absorbance was measured at 405 nm.

### B cell receptor amplification, cloning, and sequencing

Antigen-specific activated B cells were selected for B cell receptor sequence analysis. mRNA extraction, cDNA, and nested PCR reactions were done as previously described(46). Briefly, frozen cells were thawed, lysed, and mRNA extracted using TurboCapture 96 mRNA kit (QIAGEN, cat# 72251) according to the manufacturer’s protocol. mRNA was reverse transcribed, and cDNA was subjected to nested PCR reactions for Ig heavy and light chain variable regions. Amplified PCR products were analyzed with 2% 96 E-gel (Thermofisher, cat# G720802), and wells with PCR products corresponding to Ig heavy and light chain were cleaned with SPRI beads (Beckman Coulter, cat# B23319). Cleaned PCR products were sequenced directly and/or selected for Gibson cloning (NEB, cat# E2621X) prior to Sanger sequencing.

### Bioinformatic analysis of BCR sequences

#### Sequence Processing

The output from single-cell sorting Sanger sequences and 10X VDJ contigs were re-annotated with the SADIE library (https://github.com/jwillis0720/sadie.git) resulting in a paired AIRR compliant dataframe (https://www.frontiersin.org/articles/10.3389/fimmu.2018.02206/full). The dataframe was split into IGH, IGL and IGK assigned locus and paired on 10X hashtag and animal ID if they had exactly one heavy IGH and one IGL or IGK call. We also assigned the closest human ortholog to every rhesus germline V and J sequence. Somatic hypermutation was calculated by taking the number of mutations of the V_H_ or V_K_/V_L_ gene and divided by the total length of the V_H_ or V_K_/V_L_ gene.

#### BG18 Type I Definitions

The paired dataframe was assigned BG18 type I definitions as >= 22 HCDR3 amino acids long using the regular expression “ITIFG[LV]VI[IT]". Each sequence was scored based on how close it was to a perfect regular expression match and the “best” regular expression was recorded. A BG18 type I precursor was defined if it had less than four mutations from perfect regular expression match and was found in index position 4,5 or 6 in the HCDR3 sequence. In addition, we recorded if a glutamate followed +2 positions from the end of the matching regular expression.

#### BG18 Type I Alternate Definitions

The paired dataframe sequences were assigned a BG18 type I alternate classification if the sequences were >= 22 HCDR3 and had the following regular expression [WFY]GVLQFLEWLLY where up to four somatic mutations were tolerated in only VLQFLEWLLY requiring a strict regular expression match to the [WFY]G.

#### Clustering of BG18 Type I and Type 1 Alternate BCR Sequences

The clustering module of SADIE library (https://github.com/jwillis0720/Sadie.git) was used to cluster both the BG18 Type I and Type I alternate using the following criteria. The sequences were only clustered on the heavy chain and were first grouped by animal and HCDR3 length. Within each group, a distance was computed for all antibodies. The distance was calculated as a Levenshtein distance between the HCDR1s + HCDR2s + HCDR3s. In addition, the somatic pad option was used in SADIE which a distance of 1 was subtracted for every common somatic amino acid mutation (12). The final distance matrix was used for agglomerative clustering using average linkage and a distance cutoff of 3. The final clusters were annotated in the dataframe.

#### Clustering of off-target Non-BG18 Sequences

The clustering module of SADIE was also used in the clustering of off-target sequences defined as those sequences with an N332-GT5 KO antigen count of <100 (12). These sequences had distance matrix constructed such that both heavy and light chain were considered where the distance between every antibody was computed across all six CDR chains. Single-linkage agglomerative clustering was used with a cutoff of 5 to get final cluster assignments. Large clusters that were found in multiple animals and multiple weeks were prioritized for synthesis and testing with SPR.

### TZM-bl pseudovirus neutralization assay

Pseudoviruses were produced in HEK293T cells (RRID:CVCL_0063) co-transfected using FuGENE 6 (Promega, cat# E2691) with pseudovirus Env-expressing plasmid and Env-deficient backbone plasmid (PSG3ΔEnv). Pseudoviruses were harvested 72 hours post-transfection, sterile filtered (0.45 μm), and concentrated (EMD Millipore, cat# UFC905024). Equal volumes of serially diluted monoclonal antibodies at appropriate concentrations were incubated with HIV pseudovirus in half-area 96-well plates (Greiner, cat# 675083) at 37°C for 1 hour. Next, 50 μL of TZM-bl cells at 200,000 cells/mL with or without DEAE-dextran (5 μg/mL final concentration) were added to each well containing the antibody-virus mixture and incubated at 37°C for 72 hours in a humidified atmosphere of 5% CO_2_. After incubation, culture media was removed, and cells were lysed with 45 μL/well 1x Luciferase Culture Lysis buffer (Promega, cat# E1531) for 20 min at RT. Neutralization was measured by adding 30 μL luciferase reagent/well (Promega, cat# E1500) and measuring luminescence. IC_50_ was calculated using a nonlinear regression curve fit, sigmoidal, 4PL equation constrained from 0-100% in GraphPad Prism 9.3.1. IC_50_ is reported as the mean IC_50_ ± SD of two biological replicates.

### Protein expression and purification

The N332-GT5 trimer immunogen contained two modifications compared to what has been described previously (6) (fig. S18). First, the trimer was stabilized with a set of mutations called MD65, which is defined as the MD39 stabilizing mutations (5) plus four additional stabilizing mutations (V505T, V513A, V518S, L520D). Second, glycosylation sequons were added to fill the 241 and 289 glycan holes as described previously (47). Trimers in the pHLsec vector were co-transfected with furin in a 2:1 ratio into 293F cells (RRID:CVCL_D603) cultured in FreeStyle media using either 293Fectin or PEI as a transfection reagent. Proteins were harvested from the supernatant after 7 days incubation at 37°C and untagged trimers were purified by 2G12 antibody affinity chromatography using a HiTrap NHS-activated HP column (Cytiva, Cat#17-0717-01) run on an ÄKTA Pure 25L high-performance liquid chromatography (HPLC) machine (Cytiva, Cat# 29-0182-24). C-terminal His-tagged trimers were purified using a HIS-TRAP column, starting with a wash buffer (20mM Imidazole, 500 mM NaCl, 20mM Na_2_HPO_4_) and mixing in elution buffer (500 mM Imidazole, 500 mM NaCl, 20 mM Na_2_HPO_4_) using a linear gradient. Trimers were polished by size exclusion chromatography (SEC) using a Superdex 200 16/600 size exclusion chromatography column (Cytiva, Cat 28-9893-35) run on an ÄKTA Pure 25L HPLC. Final proteins were diluted in 1x TBS and stored at −80°C. For biotinylated probes, proteins were expressed with a His-tag and avi-tag (GTKHHHHHHGGSGGSGLNDIFEAQKIEWHE), purified using a HIS-TRAP column followed by SEC, and biotinylated using a BirA biotin-protein ligase reaction kit (Avidity, Cat# BirA500) according to the manufacturer instructions. The N332-GT5 and N332-GT5-KO sorting probes did not contain the 241/289 glycosylation sequons.

### Fab and antibody purification

Paired HC and LC Fab variable region sequences from select NHP affinity matured mAbs were gene synthesized and inserted into human Fab HC constant region expressing vector pFabCW and human lambda or kappa expressing vectors pCW-CLig-hL2 or pCW-CLig-hk, respectively. Fabs were expressed in 500 mL FreeStyle^™^ 293F cell cultures or 30 mL ExpiCHO^™^ cell cultures (Thermo Fisher Scientific, Cat# A29133). For 293F cell transfection, 300 μg of HC and 150 μg of LC plasmids were mixed with 225 μg polyethylenimine (PEI; 1:3 DNA:PEI ratio) in 5 mL of Opti-MEM^™^ reduced serum medium (Thermo Fisher Scientific, Cat# 31985070) for 30 min, then added to 293F cells. Supernatant was collected after 5–6 days. ExpiCHO^™^ cell cultures were transfected according to manufacturer instructions, using 12.5 μg HC and 31.2 μg LC plasmids. Supernatant was collected 8 days post transfection. Harvested supernatants were filtered through 0.45 or 0.25 μm membrane filters and batch bound to CaptureSelect CH1-XL Affinity resin (Thermo Fisher Scientific, Cat# 1943462005). Resin was washed with PBS, and captured Fabs were eluted with 50 mM NaOAc pH 4.0, buffer exchanged into 1× PBS, and concentrated using a 30k MWCO concentrator. For expression of IgG the HC variable region was cloned into the pCW-CHIg-hG1 vector. Transfection was carried out as described above but batch binding occurred overnight at 4°C to Protein A resin (Thermo Fisher Scientific, Cat# 20334) while on a rocker. Unbound supernatant was allowed to flowthrough, and the resin was washed with PBS until protein A280 reading of the flowthrough measured by a nanodrop reached background levels. Protein A bound IgG was eluted with 0.1 M Glycine pH 2.7. Eluted mAbs were buffer exchanged into 1× PBS and concentrated using a 50K MWCO concentrator (Millipore).

### SPR

Kinetics and affinities of antibody-antigen interactions were measured on a ProteOn XPR36 (Bio-Rad) using HC30M XanTec chips and 1×HBS-EP+ pH 7.4 running buffer (20× stock from Teknova, Cat. No. H8022) supplemented with BSA at 1 mg/ml. Two different capture antibodies were used: Anti-Human IgG (Fc) antibody (Cat. No. BR-1008-39, GE) for capturing IgG (ligand) at low density and flowing trimer as analyte (table S1), and His-tag Antibody (pAb, Rabbit, Cat. No. A00174, GenScript) for capturing His-tagged trimer (ligand) and flowing Fab as analyte (Figs. 3E-F and 4B, and table S4). About 7,000 response units of capture antibody were covalently immobilized on the sensor surface via EDC/NHS. In case of IgG-antigen interaction studies about 50 to 100 RUs of IgGs at 0.1ug/mL were captured onto each flow cell. In case of Fab-antigen interaction studies about 300 to 400 RUs of antigen at 1 μg/mL were captured onto each flow cell. Analytes were passed over the flow cell at 30 μl/min for 3 min followed by a 5-min dissociation time. Regeneration was accomplished using phosphoric acid 1.7% or 0.85% with a 180-s contact time and injected four times per cycle. ProteOn Manager software (Bio-Rad) was used to analyze raw sensograms, including interspot and column double referencing, and to perform either Equilibrium fits or Kinetic fits with Langmuir model, or both, when applicable.

### Cryo-electron microscopy

For each complex, 0.2 mg of N332-GT5 were incubated with 0.3 mg of BG18-like Fab (from this study) and 0.3 mg of base-directed RM20A3 Fab (to increase angular sampling in cryo-EM). A total of 5 complexes were produced (including RM_N332_03, RM_N332_36, RM_N332_32, RM_N332_07, or RM_N332_08), incubated overnight at room temperature, and purified the following morning using a HiLoad 16/600 Superdex 200 pg (Cytiva) gel filtration column. The complexes were then concentrated to between 5-6 mg/mL for application onto cryoEM grids. From a separate study, N332-GT5 was incubated with mouse polyclonal Fabs, purified as above and concentrated to 2.6 mg/ml. Later data processing (below) revealed that a majority of trimers were unliganded. Cryo grids were prepared using a Vitrobot Mark IV (Thermo Fisher Scientific). The temperature was set to 4°C and humidity was maintained at 100% during the freezing process. The blotting force was set to 1 and wait time was set to 10 s. Blotting time was varied from 5 to 6 s. Detergents lauryl maltose neopentyl glycol (LMNG; Anatrace) or n-Dodecyl-β-D-Maltoside (DDM; Anatrace) at final concentrations of 0.005 or 0.06 mM, respectively, were used for freezing. Quantifoil R 1.2/1.3 (Cu, 300-mesh; Quantifoil Micro Tools GmbH) or UltrAuFoil 1.2/1.3 (Au, 300-mesh; Quantifoil Micro Tools GmbH) grids were used and treated with Ar/O_2_ plasma (Solarus plasma cleaner, Gatan) for 8 sec before sample application. 0.5 μL of detergent was mixed with 3.5 μL of samples and 3 μL of the mixture was immediately loaded onto the grid. Following blotting, the grids were plunge-frozen into liquid nitrogen-cooled liquid ethane.

Samples containing RM_N332_03, RM_N332_36 or RM_N332_32 were loaded into a Thermo Fisher Scientific Talos Arctica operating at 200 kV. Exposure magnification was set to 36,000x with a pixel size at the specimen plane of 1.15 Å. Leginon software (48) was used for automated data collection. Micrograph movie frames were motion corrected and dose weighted using MotionCor2 (49) and imported into cryoSPARC (50) for the remainder of data processing. CTF correction was performed using cryoSPARC Patch CTF.

Samples containing RM_N332_07, or RM_N332_08 were loaded into a Thermo Fisher Scientific Glacios electron microscope operating at 200 kV. Exposure magnification was set to 190,000x with a pixel size at the specimen plane of 0.725 Å. EPU software (Thermo Fisher Scientific) was used for automated data collection. Micrograph movie frames were motion and CTF corrected using cryoSPARC Live, including dose weighting, followed by micrograph import into cryoSPARC.

N332-GT5 (partially complexed with mouse polyclonal antibodies) data collection occurred at the Pacific Northwest Center for Cryo-EM (PNCC) using a Thermo Fisher Scientific Krios and a Gatan K3 direct electron detector (300 keV, 0.40075 Å/pixel super-resolution mode). EPU (Thermo Fisher) was used for automated data collection, and Relion 3.1 (51) for motion correction Micrographs were binned during motion correction, with a resulting pixel size of 0.8015 Å/pixel and imported in cryoSPARC. CTF correction was performed using cryoSPARC Patch CTF.

For all datasets, particle picking was performed using blob picker initially followed by template picker. For Glacios datasets, particles were downscaled to 1.044 Å/pix during extraction to reduce box size and increase speed of downstream jobs. Arctica datatsets were processed at the native 1.15 Å/pix size, and the Krios dataset was processed at the binned 0.8015 Å/pix size. Multiple rounds of 2D classification and 3D ab-initio reconstruction were performed prior to 3D non-uniform refinement with global CTF refinement. For the unliganded N332-GT5 dataset, many rounds of 2D classification were performed to remove the subpopulation of particles with mouse polyclonal Fab. Final refinements were performed with C3 symmetry and global resolution estimated by FSC 0.143. Final data collection and processing stats for each dataset are summarized in table S3.

Fab Fv homology models were generated using SAbPred ABodyBuilder-ML (52). Model building was performing by docking homology models of trimer and Fab Fv in UCSF Chimera (53), manually building and refinement in Coot 0.9.8 (54) and real space refinement using Rosetta (55) and Phenix (56). Final models were validated using MolProbity and EMRinger in the Phenix suite, and statistics are summarized in table S3. All maps and models have been deposited to the Electron Microscopy Data Bank and Protein Data Bank, respectively with accession codes summarized in table S3.

### Angle of approach measurements

The trimer 3-fold axis was aligned on the z-axis with the center of mass of the CA residues of the N332 epitope on the x-axis. The center of mass for the CA residues in the N332 epitope was defined by residues G324, D325, V326, R327, M328, A329, H330, I415, L416, and P417 and was aligned on the x-axis to coordinates (40.091, 0, 0). The center of mass for three N332 epitopes at the trimer 3-fold axis was aligned to coordinates (0, 0, 0) and a third point (the center of mass for the CA of L587 in all three protomers) was aligned to coordinates (0, 0, −50.88). The latitudinal angle was the angle formed by the z-axis and a vector from the N332 epitope center of mass to the HC center of mass (CA atoms for 6 beta strands, residues 21-24, 34-39, 46-52, 67-71, 77-82, 89-92 for BG18_iGL_0_) in the x-z plane. The longitudinal angle was the angle formed by the x-axis and the same vector connecting the N332 epitope to the HC in the x-y plane. The HC-LC twist angle was the angle between the x-axis and a vector connecting the HC center of mass to the LC center of mass (CA atoms for 6 beta strands, residues 18-24, 34-37, 45-48, 62-66, 70-76, 84-88 for BG18_iGL_0_) in the x-y plane.

### Design of trimers with improved V1 loop glycan occupancy

BG505_B23 was found to have low glycan occupancy by mass spectrometry analysis and therefore three new trimers were designed using three different approaches. In the first approach, BG505_B38 was designed by reverting four V1 loop amino acids from BG505_B23 back to the WT BG505 amino acid. BG505 SOSIP has been shown to have better glycan occupancy in the V1 loop than what we observed with BG505_B23, which indicated that the germline targeting mutations in the V1 loop were causing reduced glycan occupancy (57). Therefore, reverting more GT mutations back to the WT amino acid should potentially improve the glycosylation. In the second approach, BG505_B46 was designed using the NetNGlyc server (58) to optimize the sequence adjacent to the N133 and N137 glycosylation sites. The N-terminal portion of the BG505_B23 sequence was submitted along with mutated versions containing all 20 AA at each of the following V1 loop positions: 132-136, 138, 140-143. Five V1 loop sequences were designed based on the output glycosylation potential and tested by incorporating those V1 loop sequences into the BG505_B23 trimer. They were screened for acceptable expression levels in 293F cells, SECMALS profiles to determine percent trimer, and binding affinities to BG18 type I Fabs from wks 7 and 10 post prime. Two of the trimers passed those filters and were then subjected to site-specific glycan analysis to determine glycan occupancy. Of the two trimers, BG505_B46, reported here, had a superior binding profile to BG18 type I Fabs. In the third approach, BG505_B48 was designed to have improved N137 glycan occupancy using an optimize sequence described previously (59) that modifies residues adjacent to the glycosylation site but in this case removes the N133 glycosylation site due to its proximity to N137. Therefore, BG505_B48 does not contain an N133 glycosylation sequon. All three approaches produced trimers with near complete glycan occupancy in the V1 loop when expressed in 293F cells (fig. S11). Amino acid sequences of BG505_B23, BG505_B46, and BG505_B48 are provided in fig. S18.

### Site-specific glycan analysis

#### Method 1:

DeGlyPHER (60) was used to ascertain site-specific glycan occupancy and processivity on the examined glycoproteins.

#### Proteinase K treatment and deglycosylation:

HIV Env glycoprotein was exchanged to water using Microcon Ultracel PL-10 centrifugal filter. Glycoprotein was reduced with 5 mM tris(2-carboxyethyl)phosphine hydrochloride (TCEP-HCl) and alkylated with 10 mM 2-Chloroacetamide in 100 mM ammonium acetate for 20 min at room temperature (RT, 24°C). Initial protein-level deglycosylation was performed using 250 U of Endo H for 5 μg trimer, for 1 h at 37°C. Glycorotein was digested with 1:25 Proteinase K (PK) for 30 min at 37°C. PK was denatured by incubating at 90°C for 15 min, then cooled to RT. Peptides were deglycosylated again with 250 U Endo H for 1 h at 37°C, then frozen at −80°C and lyophilized. 100 U PNGase F was lyophilized, resuspended in 20 μl 100 mM ammonium bicarbonate prepared in H_2_^18^O, and added to the lyophilized peptides. Reactions were then incubated for 1 h at 37°C, subsequently analyzed by LC-MS/MS.

#### LC-MS/MS:

Samples were analyzed on an Q Exactive HF-X mass spectrometer. Samples were injected directly onto a 25 cm, 100 μm ID column packed with BEH 1.7 μm C18 resin. Samples were separated at a flow rate of 300 nL/min on an EASY-nLC 1200 UHPLC. Buffers A and B were 0.1% formic acid in 5% and 80% acetonitrile, respectively. The following gradient was used: 1–25% B over 160 min, an increase to 40% B over 40 min, an increase to 90% B over another 10 min and 30 min at 90% B for a total run time of 240 min. Column was re-equilibrated with solution A prior to the injection of sample. Peptides were eluted from the tip of the column and nanosprayed directly into the mass spectrometer by application of 2.8 kV at the back of the column. The mass spectrometer was operated in a data dependent mode. Full MS1 scans were collected in the Orbitrap at 120,000 resolution. The ten most abundant ions per scan were selected for HCD MS/MS at 25 NCE. Dynamic exclusion was enabled with exclusion duration of 10 s and singly charged ions were excluded.

#### Data Processing:

Protein and peptide identification were done with Integrated Proteomics Pipeline (IP2). Tandem mass spectra were extracted from raw files using RawConverter (61) and searched with ProLuCID (62) against a database comprising UniProt reviewed (Swiss-Prot) proteome for Homo sapiens (UP000005640), UniProt amino acid sequences for Endo H (P04067), PNGase F (Q9XBM8), and Proteinase K (P06873), amino acid sequences for the examined proteins, and a list of general protein contaminants. The search space included no cleavage-specificity. Carbamidomethylation (+57.02146 C) was considered a static modification. Deamidation in presence of H_2_^18^O (+2.988261 N), GlcNAc (+203.079373 N), oxidation (+15.994915 M) and N-terminal pyroglutamate formation (−17.026549 Q) were considered differential modifications. Data was searched with 50 ppm precursor ion tolerance and 50 ppm fragment ion tolerance. Identified proteins were filtered using DTASelect2 (63) and utilizing a target-decoy database search strategy to limit the false discovery rate to 1%, at the spectrum level (64). A minimum of 1 peptide per protein and no tryptic end per peptide were required and precursor delta mass cut-off was fixed at 15 ppm. Statistical models for peptide mass modification (modstat) were applied. Census2 (65) label-free analysis was performed based on the precursor peak area, with a 15 ppm precursor mass tolerance and 0.1 min retention time tolerance. “Match between runs” was used to find missing peptides between runs. Data analysis using GlycoMSQuant (60) was implemented to automate the analysis. GlycoMSQuant summed precursor peak areas across replicates, discarded peptides without NGS, discarded misidentified peptides when N-glycan remnant-mass modifications were localized to non-NGS asparagines and corrected/fixed N-glycan mislocalization where appropriate.

#### Method 2:

Glycan structure identification was employed to detect specific glycoforms.

Three aliquots of each sample were denatured for 1h in 50 mM Tris/HCl, pH 8.0 containing 6 M of urea and 5 mM dithiothreitol (DTT). Next, Env proteins were reduced and alkylated by adding 20 mM iodoacetamide (IAA) and incubated for 1h in the dark, followed by a 1h incubation with 20 mM DTT to eliminate residual IAA. The alkylated Env proteins were buffer-exchanged into 50 mM Tris/HCl, pH 8.0 using Vivaspin columns (3 kDa) and two of the aliquots were digested separately overnight using trypsin, chymotrypsin (Mass Spectrometry Grade, Promega) or alpha lytic protease (Sigma Aldrich) at a ratio of 1:30 (w/w). The next day, the peptides were dried and extracted using C18 Zip-tip (MerckMilipore). The peptides were dried again, re-suspended in 0.1% formic acid and analyzed by nanoLC-ESI MS with an Ultimate 3000 HPLC (Thermo Fisher Scientific) system coupled to an Orbitrap Eclipse mass spectrometer (Thermo Fisher Scientific) using stepped higher energy collision-induced dissociation (HCD) fragmentation. Peptides were separated using an EasySpray PepMap RSLC C18 column (75 μm × 75 cm). A trapping column (PepMap 100 C18 3μM 75μM x 2cm) was used in line with the LC prior to separation with the analytical column. The LC conditions were as follows: 280 minute linear gradient consisting of 4-32% acetonitrile in 0.1% formic acid over 260 minutes followed by 20 minutes of alternating 76% acetonitrile in 0.1% formic acid and 4% Acn in 0.1% formic acid, used to ensure all the sample had eluted from the column. The flow rate was set to 300 nL/min. The spray voltage was set to 2.5 kV and the temperature of the heated capillary was set to 55 °C. The ion transfer tube temperature was set to 275 °C. The scan range was 375–1500 m/z. Stepped HCD collision energy was set to 15, 25 and 45% and the MS2 for each energy was combined. Precursor and fragment detection were performed using an Orbitrap at a resolution MS1= 120,000. MS2= 30,000. The AGC target for MS1 was set to standard and injection time set to auto which involves the system setting the two parameters to maximize sensitivity while maintaining cycle time.

Glycopeptide fragmentation data were extracted from the raw file using Byos (Version 3.5; Protein Metrics Inc.). The MS data was searched using the Protein Metrics 305 N-glycan library with sulfated glycans added manually. The relative amounts of each glycan at each site as well as the unoccupied proportion were determined by comparing the extracted chromatographic areas for different glycotypes with an identical peptide sequence. All charge states for a single glycopeptide were summed. The precursor mass tolerance was set at 4 ppm and 10 ppm for fragments. A 1% false discovery rate (FDR) was applied. The relative amounts of each glycan at each site as well as the unoccupied proportion were determined by comparing the extracted ion chromatographic areas for different glycopeptides with an identical peptide sequence. Glycans were categorized according to the composition detected.

HexNAc(2)Hex(10+) was defined as M9Glc, HexNAc(2)Hex(9–5) was classified as M9 to M3. Any of these structures containing a fucose were categorized as FM (fucosylated mannose). HexNAc(3)Hex(5–6)X was classified as Hybrid with HexNAc(3)Hex(5-6)Fuc(1)X classified as Fhybrid. Complex-type glycans were classified according to the number of HexNAc subunits and the presence or absence of fucosylation. As this fragmentation method does not provide linkage information compositional isomers are grouped, so for example a triantennary glycan contains HexNAc 5 but so does a biantennary glycans with a bisect. Core glycans refer to truncated structures smaller than M3. M9glc- M4 were classified as oligomannose-type glycans. Glycans containing at least one sialic acid were categorized as NeuAc and glycans containing at least one fucose residue were categorized as fucose.

## Supplementary Material

supplementalMaterial
