# Supplementary material for: Vaccine priming of rare HIV broadly neutralizing antibody precursors in non-human primates
Source: Science. Author manuscript; Available in PMC 2024 Aug 8. (PMC11309785; doi:10.1126/science.adj8321)
Supplement: supplementalMaterial [file NIHMS2008143-supplement-supplementalMaterial.docx]

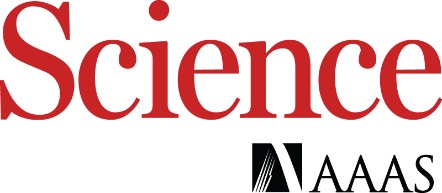


Supplementary Materials for

Vaccine priming of rare HIV broadly neutralizing antibody precursors in non-human primates

Jon M Steichen, Ivy Phung, Eugenia Salcedo, Gabriel Ozorowski, Jordan R. Willis, Sabyasachi Baboo, Alessia Liguori, Christopher A. Cottrell, Jonathan L. Torres, Patrick J. Madden, Krystal M. Ma, Henry J. Sutton, Jeong Hyun Lee, Oleksandr Kalyuzhniy, Joel D. Allen, Oscar L. Rodriguez, Yumiko Adachi, Tina-Marie Mullen, Erik Georgeson, Michael Kubitz, Alison Burns, Shawn Barman, Rohini Mopuri, Amanda Metz, Tasha K. Altheide, Jolene K. Diedrich, Swati Saha, Kaitlyn Shields, Steven E. Schultze, Melissa L. Smith, Torben Schiffner, Dennis R. Burton, Corey T. Watson, Steven E. Bosinger, Max Crispin, John R. Yates III, James C. Paulson, Andrew B. Ward^*^, Devin Sok^*^, Shane Crotty^*^, William R. Schief^*^

Corresponding authors: Andrew B. Ward, [andrew@scripps.edu](http://andrew@scripps.edu); Devin Sok, [dsok@iavi.org](http://dsok@iavi.org); Shane Crotty, [shane@lji.org](http://shane@lji.org); and William R. Schief, [schief@scripps.edu](http://schief@scripps.edu)

**This PDF file includes:**

Figs. S1 to S18

Tables S1 to S5

**Fig.** **S1. Comparison of BG18 precursor frequency in macaques and humans.** (A) Comparison of HCDR3 length distributions from human BCRs (*6, 39*) and macaques IgMs shows that humans have a higher frequency of longer HCDR3s. (B) HCDR3 length distribution separated by sequencing method. Human, all human BCR HC sequences from (*6, 39*); RM all, 98 datasets of rhesus macaque BCR HC sequences from multiple studies (see methods); RM all IgM (not this study), IgM sequences from “RM all” with the 4 data sets from this study removed; RM 5’ RACE IgMs, RM IgM sequences using 5’RACE and constant region primers; RM 5’UTR IgMs, RM IgM sequences using 5’UTR primers and an IgM constant region primer; RM SP IgMs, RM IgM sequences using signal peptide region primers and constant region primers; RM (this study), the 4 animals that were sequenced in this study. (C) Difference in D gene usage frequency between humans and macaques shows a slightly higher frequency of D3-3 usage in the human repertoire compared to D3-41 usage in macaques. (D) Comparison of frequencies between humans and macaque naïve sequences across multiple sequence definitions show that NHPs have lower frequencies of BG18-like precursors at all levels. (E) Criteria used to search for BG18 precursors in humans and RMs.

**Fig. S2. ELISA response.** (A) Schematic of vaccination and time points of plasma collection. ELISA area under the curve showing plasma response at the indicated time points to (B) N332-GT5 trimer or (C) N332-GT5-KO captured by C-terminal His-tag. (D) The difference between the N332-GT5 and N332-GT5-KO responses. (E) Area under the curve ELISA response to the MD39-V3 peptide. Area under the curve ELISA response to (F) BG505 SOSIP MD39 or (G) BG505 SOSIP MD39 BB with mutations that block binding to the bottom of the trimer captured by PGT128 Fab. (H) The difference in area under the curves between MD39 and MD39 BB showing plasma response to the bottom of the trimer. For B-H, lines indicate the median with interquartile range (n=8).

**
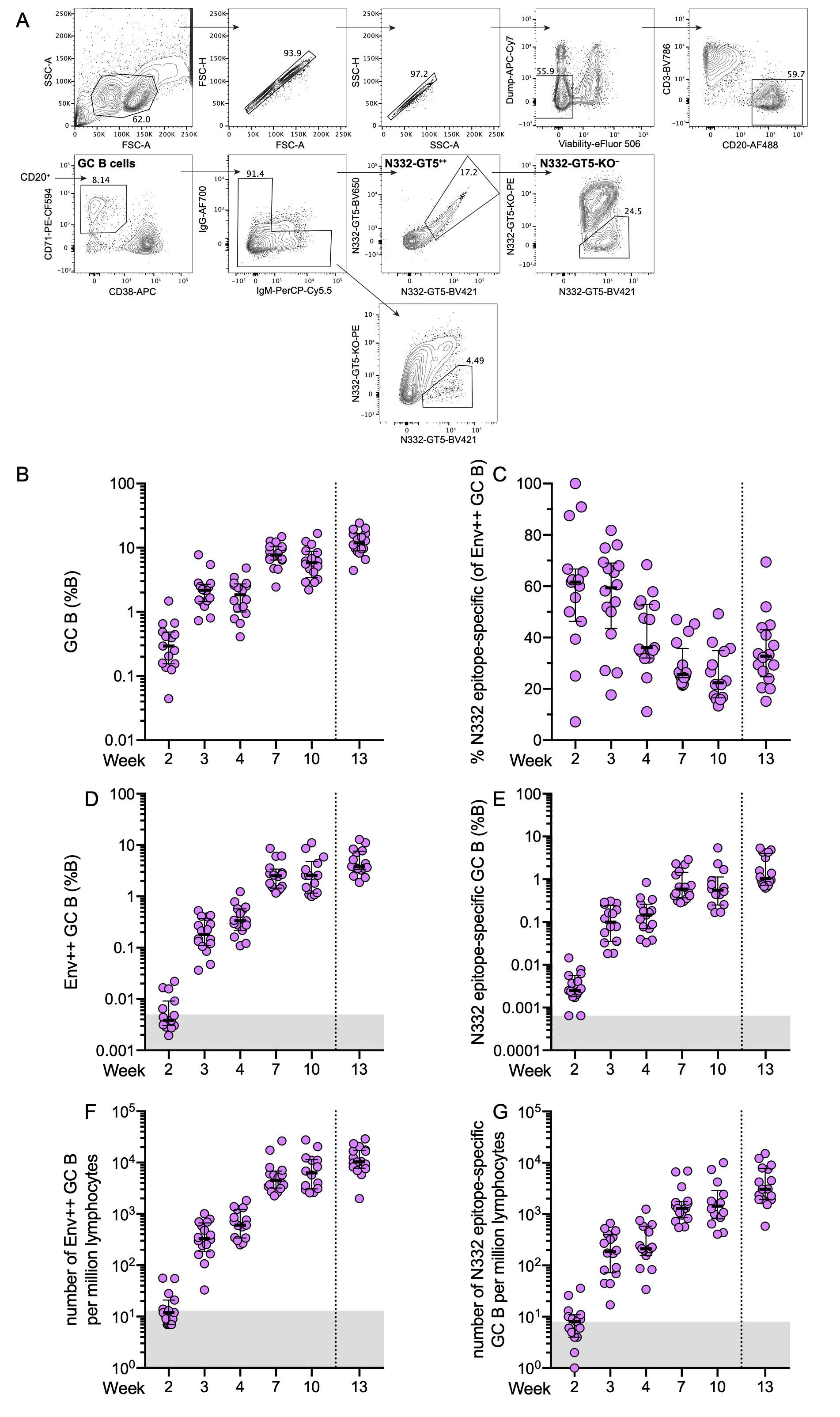
**

**Fig. S3. Analysis of GC B cells.** (A) Gating strategy for longitudinal analysis of GC B cells. N332-GT5-binding B cells are gated as N332-GT5-BV650^+^ / N332-GT5-BV421^+^ dual binders, and termed “N332-GT5^++^” or “Env^++^” hereafter. (B) Quantification of GC B cell kinetics. The dotted line separates post-prime and post-boost timepoints. (C) Frequency of N332 epitope-specific (Env^++^KO^-^) GC B cells among Env^++^ GC B cells. (D) Frequency of Env^++^ GC B cells as a percentage of total CD20^+^ B cells. The gray area is set from 0.001% to the median frequency of Env^++^ GC B cells observed in the pre-immunization samples. (E) Frequency of N332 epitope-specific GC B cells as a percentage of total CD20^+^ B cells. The gray area is set from 0.0001% to calculated limit of detection. (F) Quantification of Env^++^ GC B cells per million lymphocytes recorded. They gray area is set from 1 to the median number of epitope-specific GC B cells observed in the pre-immunization samples. (G) Number of N332 epitope-specific GC B cells per million lymphocytes recorded. The gray area is set from 1 to the median number of epitope-specific GC B cells observed in the pre-immunization samples. Each circle represents a 1 mL aliquot of FNA cells. Data from FNAs from both the left and right side are graphed individually for each animal. Lines indicate the median with interquartile range.

**Fig. S4. Memory B cells sorting.** (A) Gating strategy for analysis of week 10 IgD^-^ memory B cells. (B) Antigen-specific (N332-GT5-AF647^+^N332-GT5-BV421^+^) memory B cells (CD20^+^IgD^–^) from Fig. S4A were backgated to assess surface immunoglobulin (Ig) expression by IgD versus IgG MFI signals. The gate revealed that most of the antigen-specific memory B cells expressed IgG, indicating successful identification of antigen-specific memory B cells. (C) Representative flow plot of antigen-specific memory B cells (gated as in Figure S4A) from a pre-immunization PBMC sample compared to the post-immunization timepoint (week 12) from the same animal, confirming specificity of N332-GT5 and N332-GT5-KO probe staining. (D) Gating strategy for analysis of week 12 IgG^+^ memory B cells. (E) Frequencies of N332-GT5^++^ B cells among IgD^-^ (week 10) or IgG^+^ (week 12) memory B cells. (F) Frequency of N332 epitope-specific B cells among IgD^-^ (week 10) or IgG^+^ (week 12) memory B cells. (G) Frequency of N332 epitope-specific B cells among N332-GT5^++^ B cells in IgD^-^ (week 10) or IgG^+^ (week 12) memory B cells. For E-G, lines indicate the median with interquartile range (n = 8).

**Fig. S5. Schematic for sorting, B cell activation, functional screening and sequencing memory B cells.** (A) Overall strategy for sorting memory B cells, functional characterization and sequencing. (B) ELISA OD405 for screening supernatants of activated memory B cells for binding to N332-GT5. BG18 is a positive control mAb and Den3 is a negative control mAb.

**Fig. S6. Clonal lineage trees of BG18-like B cells.** Each tree denotes a clone, with clone ID, animal origin, and V, D, and J gene calls. Branch lengths are scaled by estimated heavy chain nucleotide mutations (1 scale unit = 5 mutations). Tree tips are colored by week of sampling. Representative heavy chain and light chain CDR3 sequences are provided for each clone.

**Fig. S7. Frequency of Glu at the (D3-41)+2 position in macaque antibodies.** (A) Frequency of glutamate positioned 2 aa past the end of the D3-41 gene when D3-41 is present in the BG18 reading frame and position (+/- 1 aa) in antibodies with HCDR3s ≥22 aa compared to antibodies with HCDR3s <22 aa and D3-41 positioned anywhere in sequences isolated from GC B cells. (B) as in (A) but sequences were derived from memory B cells.

**
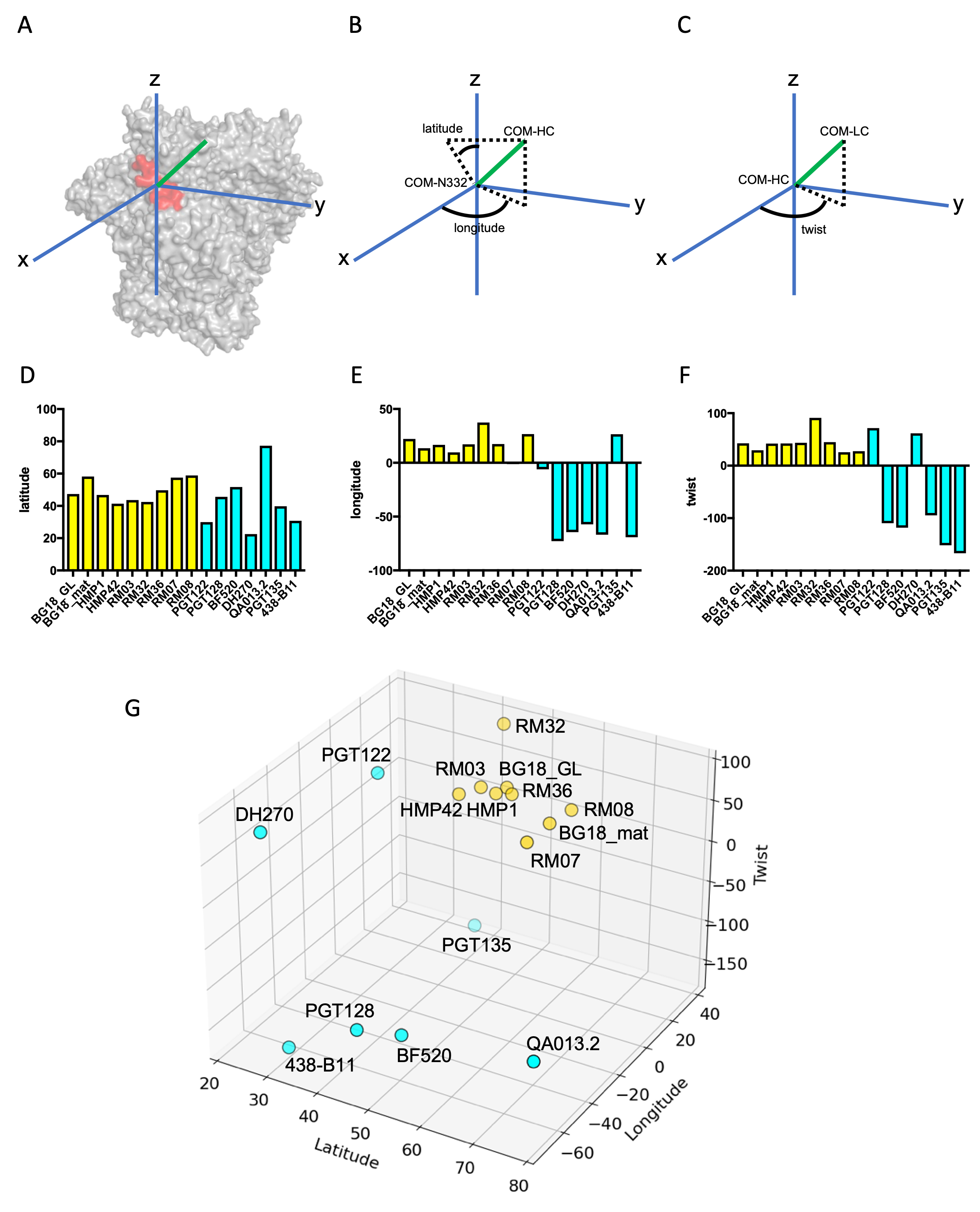
**

**Fig. S8. Angles of approach for N332/V3 glycan antibodies**. **(A)** Angle of approach (green) relative to the trimer (gray) and N332 epitope (red). **(B)** Diagram showing definition of latitude and longitude angles. **(C)** definition of HC-LC twist angle. **(D** to **F)** Latitudinal (D), longitudinal (E), and HC-LC twist angles (F) for BG18 (6DFG), BG18_iGL_0_ (6DFH), HMP1 (6NF5), HMP42 (6NFC), RM_N332_03, RM_N332_32, RM_N332_36, RM_N332_07, RM_N332_08 in yellow and seven other N332-dependent bnAbs: PGT122 (4TVP), DH270.6 (6UM6), BF520.1 (6MN7), PGT128 (5ACO), QA013.2 (7N65), PGT135 (4JM2), 438-B11 (6UTK) in cyan. **(G)** 3D scatter plot showing latitudinal, longitudinal and HC-LC twist angles for the antibodies in (D), (E), and (F).

**Fig. S9. Epitope footprints of BG18-like antibodies.** The epitope footprint for nine BG18-like antibodies (5 from this study and 4 from (*6*)) are shown in red (defined as atoms within 5 Å of the Fab). The yellow oval encompasses the footprint of BG18_GL_0_ and is mapped onto the other eight structures for comparison. gp120 is colored gray. The five structures from this study are RM_N332_36, RM_N332_03, RM_N332_32, RM_N332_07 and RM_N332_08. The four structures from (*6*) are BG18_GL0 (PDB ID: 6DFH), BG18_Mat (PDB ID: 6DFG), HMP1 (PDB ID: 6NF5) and HMP42 (PDB ID: 6NFC).

**Fig. S10. Other BG18-like antibodies.** (A) Cryo-EM structure of N332-GT2 in complex with BG18_GL_0_ (PDB ID: 6DFH). Inset shows the interactions of the HCDR3 to the conserved residues in gp120. (B) Cryo-EM structure of Fab RM_N332_07 in complex with N332-GT5. Inset shows HCDR3 interactions to gp120. (C) Cryo-EM structure of Fab RM_N332_08 in complex with N332-GT5. Inset shows HCDR3 interactions to gp120.

**Fig. S11. Glycan analysis of V1 modified trimers.** (A) V1 loop sequences of V1 loop modified trimers. All five trimers have identical sequences outside of the region shown. Differences are highlighted green. (B) Glycan analysis of V1 modified trimers. Green indicates high mannose glycans, Pink indicates complex type glycans and Gray indicates unoccupied glycosylation sites.

**Fig. S12. Site-specific glycan compositions of BG505 MD65 B23.** LC-MS analysis of the glycosylation of BG505 MD65 B23. Oligomannose-type glycans are colored green, hybrid-type in hashed pink, complex-type in pink, and the proportion of unoccupied PNGS in grey. The table represents the grouping of the bar graphs, with any glycan composition containing at least one fucose or sialic acid (NeuAc) shown.

**Fig. S13. Neutralization assay of V1 modified pseudoviruses against three BG18 type I antibodies**. Three N332-GT5 elicited Rhesus macaque antibodies were tested for neutralization activity against BG505 pseudovirus and four variants of BG505 containing the N332-GT5 V1 loop with or without adding the N133, N137, or both N133 and N137 glycosylation sequons. BG18 and PGT121 are positive controls and Den3 is a negative control.

**Fig. S14. Genetic features of non-BG18 epitope-specific BCRs isolated from weeks 7 and 10 post prime.** (A) HCDR3 lengths for 45 non-BG18-like competitor antibodies isolated from GC B cells at weeks 7 and 10. (B) VH gene usage for the non-BG18-like competitors. (C) VL gene usage for the non-BG18-like competitors.

**Fig. S15.** **Structure of immunogenic residues in unliganded N332-GT5.** The BG18 epitope on unliganded N332-GT5 is shown with solvent exposed V1 loop aa side chains (K137 and R139) that are critical for binding to non-BG18-like competitor antibodies indicated. V1 loop, green; V3 loop, yellow. The three mutations in the B23 trimer relative to N332-GT5 are A135T, K137N and R139T.

**Fig. S16. No indication of a lambda3-based BG18 type II response in rhesus macaques.** HCDR3 length distribution of antibodies that use VL3 light chains from all epitope-specific GC B cells combined with and without BG18 type I antibodies removed.

**Fig. S17. ELISA binding to activated B cell supernatants from N332-GT5+ B cells isolated 2 weeks post boost**. Each symbol represents the ELISA signal from one well and they are ordered based on the HCDR3 length of the BCR sequence identified in each well. BG18 type II antibodies would be found in wells that contained BCRs with HCDR3s ≥ 20 aa (shaded gray). The plot on top shows ELISA reactivity to N332-GT5 and N332-GT5-KO. The bottom plot shows ELISA reactivity to the B23 trimer. The BG18 type I antibodies are indicated on the plots.

BG505_MD39 AENLWVTVYYGVPVWKDAETTLFCASDAKAYETEKHNVWATHACVPTDPNPQEIHLENVT

BG505_MD39_N332-GT5 AENLWVTVYYGVPVWKDAETTLFCASDAKAYETEKHNVWATHACVPTDPNPQEIHLENVT

BG505_MD65_congly_N332-GT5 AENLWVTVYYGVPVWKDAETTLFCASDAKAYETEKHNVWATHACVPTDPNPQEIHLENVT

BG505_MD65_B38 AENLWVTVYYGVPVWKDAETTLFCASDAKAYETEKHNVWATHACVPTDPNPQEIHLENVT

BG505_MD65_B48 AENLWVTVYYGVPVWKDAETTLFCASDAKAYETEKHNVWATHACVPTDPNPQEIHLENVT

BG505_MD65_B23 AENLWVTVYYGVPVWKDAETTLFCASDAKAYETEKHNVWATHACVPTDPNPQEIHLENVT

BG505_MD65_B46 AENLWVTVYYGVPVWKDAETTLFCASDAKAYETEKHNVWATHACVPTDPNPQEIHLENVT

BG505_MD39 EEFNMWKNNMVEQMHEDIISLWDQSLKPCVKLTPLCVTLQCTNVTNNITDDMRGELKNCS

BG505_MD39_N332-GT5 EEFNMWKNNMVEQMHEDIISLWDQSLKPCVKLTPLCVTLQCTNYAPKLRSMMRGEIKNCS

BG505_MD65_congly_N332-GT5 EEFNMWKNNMVEQMHEDIISLWDQSLKPCVKLTPLCVTLQCTNYAPKLRSMMRGEIKNCS

BG505_MD65_B38 EEFNMWKNNMVEQMHEDIISLWDQSLKPCVKLTPLCVTLQCTNVTNNITSMMRGELKNCS

BG505_MD65_B48 EEFNMWKNNMVEQMHEDIISLWDQSLKPCVKLTPLCVTLQCTNYMSNGTSMMRGEIKNCS

BG505_MD65_B23 EEFNMWKNNMVEQMHEDIISLWDQSLKPCVKLTPLCVTLQCTNYTPNLTSMMRGEIKNCS

BG505_MD65_B46 EEFNMWKNNMVEQMHEDIISLWDQSLKPCVKLTPLCVTLQCTNFTLNLTSMMRGEIKNCS

BG505_MD39 FNMTTELRDKKQKVYSLFYRLDVVQINENQGNRSNNSNKEYRLINCNTSAITQACPKVSF

BG505_MD39_N332-GT5 FNMTTELRDKKQKVYSLFYRLDVVQINENQGNRSNNSNKEYRLINCNTSAITQACPKVSF

BG505_MD65_congly_N332-GT5 FNMTTELRDKKQKVYSLFYRLDVVQINENQGNRSNNSNKEYRLINCNTSAITQACPKVSF

BG505_MD65_B38 FNMTTELRDKKQKVYSLFYRLDVVQINENQGNRSNNSNKEYRLINCNTSAITQACPKVSF

BG505_MD65_B48 FNMTTELRDKKQKVYSLFYRLDVVQINENQGNRSNNSNKEYRLINCNTSAITQACPKVSF

BG505_MD65_B23 FNMTTELRDKKQKVYSLFYRLDVVQINENQGNRSNNSNKEYRLINCNTSAITQACPKVSF

BG505_MD65_B46 FNMTTELRDKKQKVYSLFYRLDVVQINENQGNRSNNSNKEYRLINCNTSAITQACPKVSF

BG505_MD39 EPIPIHYCAPAGFAILKCKDKKFNGTGPCPSVSTVQCTHGIKPVVSTQLLLNGSLAEEEV

BG505_MD39_N332-GT5 EPIPIHYCAPAGFAILKCKDKKFNGTGPCPSVSTVQCTHGIKPVVSTQLLLNGSLAEEEV

BG505_MD65_congly_N332-GT5 EPIPIHYCAPAGFAILKCKDKKFNGTGPCQNVSTVQCTHGIKPVVSTQLLLNGSLAEEEV

BG505_MD65_B38 EPIPIHYCAPAGFAILKCKDKKFNGTGPCPSVSTVQCTHGIKPVVSTQLLLNGSLAEEEV

BG505_MD65_B48 EPIPIHYCAPAGFAILKCKDKKFNGTGPCPSVSTVQCTHGIKPVVSTQLLLNGSLAEEEV

BG505_MD65_B23 EPIPIHYCAPAGFAILKCKDKKFNGTGPCPSVSTVQCTHGIKPVVSTQLLLNGSLAEEEV

BG505_MD65_B46 EPIPIHYCAPAGFAILKCKDKKFNGTGPCPSVSTVQCTHGIKPVVSTQLLLNGSLAEEEV

BG505_MD39 IIRSENITNNAKNILVQLNTPVQINCTRPNNNTVKSIRIGPGQAFYYTGDIIGDIRQAHC

BG505_MD39_N332-GT5 IIRSENITNNAKNILVQLNTPVQINCTRPSNNTVKSIRIGPGQAFYYFGDVLGHVRMAHC

BG505_MD65_congly_N332-GT5 IIRSENITNNAKNILVQLNTSVQINCTRPSNNTVKSIRIGPGQAFYYFGDVLGHVRMAHC

BG505_MD65_B38 IIRSENITNNAKNILVQLNTPVQINCTRPSNNTVKSIRIGPGQAFYYFGDVLGHVRMAHC

BG505_MD65_B48 IIRSENITNNAKNILVQLNTPVQINCTRPSNNTVKSIRIGPGQAFYYFGDVLGHVRMAHC

BG505_MD65_B23 IIRSENITNNAKNILVQLNTPVQINCTRPSNNTVKSIRIGPGQAFYYFGDVLGHVRMAHC

BG505_MD65_B46 IIRSENITNNAKNILVQLNTPVQINCTRPSNNTVKSIRIGPGQAFYYFGDVLGHVRMAHC

BG505_MD39 NVSKATWNETLGKVVKQLRKHFGNNTIIRFAQSSGGDLEVTTHSFNCGGEFFYCNTSGLF

BG505_MD39_N332-GT5 NISKATWNETLGKVVKQLRKHFGNNTIIRFAQSSGGDLEVTTHSFNCGGEFFYCNTSGLF

BG505_MD65_congly_N332-GT5 NISKATWNETLGKVVKQLRKHFGNNTIIRFAQSSGGDLEVTTHSFNCGGEFFYCNTSGLF

BG505_MD65_B38 NISKATWNETLGKVVKQLRKHFGNNTIIRFAQSSGGDLEVTTHSFNCGGEFFYCNTSGLF

BG505_MD65_B48 NISKATWNETLGKVVKQLRKHFGNNTIIRFAQSSGGDLEVTTHSFNCGGEFFYCNTSGLF

BG505_MD65_B23 NISKATWNETLGKVVKQLRKHFGNNTIIRFAQSSGGDLEVTTHSFNCGGEFFYCNTSGLF

BG505_MD65_B46 NISKATWNETLGKVVKQLRKHFGNNTIIRFAQSSGGDLEVTTHSFNCGGEFFYCNTSGLF

BG505_MD39 NSTWISNTSVQGSNSTGSNDSITLPCRIKQIINMWQRIGQAMYAPPIQGVIRCVSNITGL

BG505_MD39_N332-GT5 NSTWISNTSVQGSNSTGSNDSLILPCWIKQIINMWQRIGQAMYAPPIQGVIRCVSNITGL

BG505_MD65_congly_N332-GT5 NSTWISNTSVQGSNSTGSNDSLILPCWIKQIINMWQRIGQAMYAPPIQGVIRCVSNITGL

BG505_MD65_B38 NSTWISNTSVQGSNSTGSNDSLILPCWIKQIINMWQRIGQAMYAPPIQGVIRCVSNITGL

BG505_MD65_B48 NSTWISNTSVQGSNSTGSNDSLILPCWIKQIINMWQRIGQAMYAPPIQGVIRCVSNITGL

BG505_MD65_B23 NSTWISNTSVQGSNSTGSNDSLILPCWIKQIINMWQRIGQAMYAPPIQGVIRCVSNITGL

BG505_MD65_B46 NSTWISNTSVQGSNSTGSNDSLILPCWIKQIINMWQRIGQAMYAPPIQGVIRCVSNITGL

BG505_MD39 ILTRDGGSTNSTTETFRPGGGDMRDNWRSELYKYKVVKIEPLGVAPTRCKRRVVGRRRRR

BG505_MD39_N332-GT5 ILTRDGGSTNSTTETFRPGGGDMRDNWRSELYKYKVVKIEPLGVAPTRCKRRVVGRRRRR

BG505_MD65_congly_N332-GT5 ILTRDGGSTNSTTETFRPGGGDMRDNWRSELYKYKVVKIEPLGVAPTRCKRRTVGRRRRR

BG505_MD65_B38 ILTRDGGSTNSTTETFRPGGGDMRDNWRSELYKYKVVKIEPLGVAPTRCKRRTVGRRRRR

BG505_MD65_B48 ILTRDGGSTNSTTETFRPGGGDMRDNWRSELYKYKVVKIEPLGVAPTRCKRRTVGRRRRR

BG505_MD65_B23 ILTRDGGSTNSTTETFRPGGGDMRDNWRSELYKYKVVKIEPLGVAPTRCKRRTVGRRRRR

BG505_MD65_B46 ILTRDGGSTNSTTETFRPGGGDMRDNWRSELYKYKVVKIEPLGVAPTRCKRRTVGRRRRR

BG505_MD39 RAVGIGAVSLGFLGAAGSTMGAASMTLTVQARNLLSGIVQQQSNLLRAPEPQQHLLKDTH

BG505_MD39_N332-GT5 RAVGIGAVSLGFLGAAGSTMGAASMTLTVQARNLLSGIVQQQSNLLRAPEPQQHLLKDTH

BG505_MD65_congly_N332-GT5 RAAGIGASSDGFLGAAGSTMGAASMTLTVQARNLLSGIVQQQSNLLRAPEPQQHLLKDTH

BG505_MD65_B38 RAAGIGASSDGFLGAAGSTMGAASMTLTVQARNLLSGIVQQQSNLLRAPEPQQHLLKDTH

BG505_MD65_B48 RAAGIGASSDGFLGAAGSTMGAASMTLTVQARNLLSGIVQQQSNLLRAPEPQQHLLKDTH

BG505_MD65_B23 RAAGIGASSDGFLGAAGSTMGAASMTLTVQARNLLSGIVQQQSNLLRAPEPQQHLLKDTH

BG505_MD65_B46 RAAGIGASSDGFLGAAGSTMGAASMTLTVQARNLLSGIVQQQSNLLRAPEPQQHLLKDTH

BG505_MD39 WGIKQLQARVLAVEHYLRDQQLLGIWGCSGKLICCTNVPWNSSWSNRNLSEIWDNMTWLQ

BG505_MD39_N332-GT5 WGIKQLQARVLAVEHYLRDQQLLGIWGCSGKLICCTNVPWNSSWSNRNLSEIWDNMTWLQ

BG505_MD65_congly_N332-GT5 WGIKQLQARVLAVEHYLRDQQLLGIWGCSGKLICCTNVPWNSSWSNRNLSEIWDNMTWLQ

BG505_MD65_B38 WGIKQLQARVLAVEHYLRDQQLLGIWGCSGKLICCTNVPWNSSWSNRNLSEIWDNMTWLQ

BG505_MD65_B48 WGIKQLQARVLAVEHYLRDQQLLGIWGCSGKLICCTNVPWNSSWSNRNLSEIWDNMTWLQ

BG505_MD65_B23 WGIKQLQARVLAVEHYLRDQQLLGIWGCSGKLICCTNVPWNSSWSNRNLSEIWDNMTWLQ

BG505_MD65_B46 WGIKQLQARVLAVEHYLRDQQLLGIWGCSGKLICCTNVPWNSSWSNRNLSEIWDNMTWLQ

BG505_MD39 WDKEISNYTQIIYGLLEESQNQQEKNEQDLLALD

BG505_MD39_N332-GT5 WDKEISNYTQIIYGLLEESQNQQEKNEQDLLALD

BG505_MD65_congly_N332-GT5 WDKEISNYTQIIYGLLEESQNQQEKNEQDLLALD

BG505_MD65_B38 WDKEISNYTQIIYGLLEESQNQQEKNEQDLLALD

BG505_MD65_B48 WDKEISNYTQIIYGLLEESQNQQEKNEQDLLALD

BG505_MD65_B23 WDKEISNYTQIIYGLLEESQNQQEKNEQDLLALD

BG505_MD65_B46 WDKEISNYTQIIYGLLEESQNQQEKNEQDLLALD

**Fig. S18. Amino acid sequence alignment of trimers used in this study.** Green highlight indicates amino acid changes relative to BG505_MD39 (*5*). The immunogen used in this study, referred to as N332-GT5, is labelled BG505_MD65_congly_N332-GT5 in the sequence alignment. Congly indicates glycosylation sites at positions 241 and 289 were introduced.

**Table S1. Binding affinity of N332-GT5 to the BG18 inferred germline with various amino acids substituted into the (D3-3)+2 position.**

| **Amino acid at**  **(D3-3)+2 position** | **K_D_ (nM)** |
| --- | --- |
| Glu | 0.7 |
| Asp | 39 |
| Gly | 1790 |
| Asn | NB |
| Ser | NB |
| Ala | NB |

SPR K_D_ measured by capturing IgG at low density and flowing trimer as analyte. NB, indicates no binding was detected at 3 µM, the highest concentration tested.

**Table S2. Genotype of RMs immunized in this study.**

| **Animal** | **Genotype IGHD3-41** |
| --- | --- |
| L610 | *01/*01_S8240 |
| DHHW | *01/*01_S8240 |
| DHIC | *01/*01 |
| K397 | *01/*01 |
| K410 | *01/*01_S8240 |
| K916 | *01_S8240/*01_S8240 |
| L211 | *01/*01_S8240 |
| L611 | *01_S8240/*01_S8240 |

*01, frame 3: ITIFGLVII; frame 1: VLQYLDWLLY

*01_S8240, frame 3: ITIFGVVIT; frame 1: VLQFLEWLLH

**Table S3. Cryo-EM data collection, refinement and validation statistics**

|  | MD65 N332-GT5 + RM_N332_03 + RM20A3  (EMDB-41024)  (PDB 8t49) | MD65 N332-GT5 + RM_N332_36 + RM20A3  (EMDB-41025)  (PDB 8t4a) | MD65 N332-GT5 + RM_N332_32 + RM20A3  (EMDB-41026)  (PDB 8t4b) | MD65 N332-GT5 + RM_N332_07 + RM20A3  (EMDB-41035)  (PDB 8t4l) | N332-GT5 + RM_N332_08 + RM20A3  (EMDB-41027)  (PDB 8t4d) | MD64 N332-GT5  (EMDB-41034)  (PDB 8t4k) |
| --- | --- | --- | --- | --- | --- | --- |
| **Data collection and processing** |  |  |  |  |  |  |
| Microscope | TFS Arctica | TFS Arctica | TFS Arctica | TFS Glacios | TFS Glacios | TFS Titan Krios |
| Magnification | 36,000x | 36,000x | 36,000x | 190,000x | 190,000x | 29,000x |
| Voltage (kV) | 200 | 200 | 200 | 200 | 200 | 300 |
| Electron exposure (e–/Å^2^) | 51 | 51 | 51 | 43 | 50 | 79 |
| Defocus range (μm) | -0.5 to -2.0 | -0.5 to -2.0 | -0.5 to -2.0 | -0.5 to -1.4 | -0.5 to -1.4 | -0.8 to -2.5 |
| Detector | Gatan K2 Summit | Gatan K2 Summit | Gatan K2 Summit | TFS Falcon 4 | TFS Falcon 4 | Gatan K3 |
| Recording mode | Counting | Counting | Counting | Counting | Counting | Super-resolution |
| Pixel size (Å) | 1.15 | 1.15 | 1.15 | 0.725 | 0.725 | 0.40075 |
| Symmetry imposed | C3 | C3 | C3 | C3 | C3 | C3 |
| Micrographs (no.) | 1,922 | 2,266 | 1,970 | 3,911 | 6,010 | 7,165 |
| Initial particle images (no.) | 522,025 | 627,786 | 469,715 | 610,785 | 930,534 | 2,173,301 |
| Final particle images (no.) | 57,573 | 75,027 | 108,784 | 34,150 | 99,087 | 202,102 |
| Map resolution (Å)  FSC threshold | 3.2  0.143 | 3.4  0.143 | 3.5  0.143 | 3.2  0.143 | 3.1  0.143 | 2.6  0.143 |
| Map sharpening *B* factor (Å^2^) | -85.2 | -108.2 | -120.2 | -68.6 | -84.6 | -88.0 |
| Map pixel size (Å) | 1.15 | 1.15 | 1.15 | 1.044 | 1.044 | 0.8015 |
| Map resolution range (Å) | 2.4-5.2 | 2.5-5.5 | 2.5-5.2 | 2.4-5.2 | 2.2-5.2 | 1.8-3.0 |
|  |  |  |  |  |  |  |
| **Refinement** |  |  |  |  |  |  |
| Initial model used (PDB code) | 6nf5 | 6nf5 | 6nf5 | 6nf5 | 6nf5 | 6nf5 |
| Model resolution (Å)  FSC threshold | 3.3  0.5 | 3.6  0.5 | 3.6  0.5 | 3.5  0.5 | 3.2  0.5 | 2.7  0.5 |
| Model resolution range (Å) | 2.4-4.0 | 2.5-4.0 | 2.5-3.7 | 2.4-3.6 | 2.2-4.2 | 1.8-3.0 |
| EMRinger score | 3.95 | 3.26 | 3.37 | 2.88 | 4.05 | 5.26 |
| Model composition  Non-hydrogen atoms  Protein residues  Ligands | 24,939  3,045  96 | 24,924  3,057  81 | 24,525  3,039  69 | 24,375  3,039  57 | 24,756  3,060  63 | 13,560  1,638  39 |
| Mean *B* factors (Å^2^)  Protein  Ligand | 81.76  87.68 | 95.56  84.99 | 65.51  60.98 | 88.60  90.47 | 53.21  50.37 | 37.86  41.26 |
| R.m.s. deviations  Bond lengths (Å)  Bond angles (°) | 0.006  1.103 | 0.007  1.108 | 0.006  1.037 | 0.006  1.016 | 0.005  0.980 | 0.007  1.070 |
| Validation  MolProbity score  Clashscore  Poor rotamers (%) | 0.92  1.53  0.57 | 0.85  1.16  0.23 | 0.96  1.08  0.34 | 1.03  2.40  0.34 | 0.94  1.39  0.23 | 0.80  1.04  0.62 |
| Ramachandran plot  Favored (%)  Allowed (%)  Disallowed (%) | 97.89  2.11  0.00 | 97.89  2.11  0.00 | 97.17  2.83  0.00 | 97.98  2.02  0.00 | 97.70  2.30  0.00 | 98.87  0.94  0.19 |

**Table S4. Potential human D genes for BG18 type I antibodies.**

| Antibody | D gene | Reading frame | N332-GT5 K_D_ nM | HCDR3 |
| --- | --- | --- | --- | --- |
| PGT121 | D3-3*01 | 3 |  | ARTLHGRRIYGIVAFNEWFTYFYMDV |
| BG18 | D3-3*01 | 3 |  | ARNAIRIYGVVALGEWFHYGMDV |
| BG18-GL_0_ | D3-3*01 | 3 | 0.4 | ARNAITIFGVVIIGEYYYYGMDV |
| BG18-GL_Alt1_ | D3-3*02 | 1 | 0.023 | ARNAIRIYGVLAFLEWLLYGMDV |
| BG18-GL_Alt2_ | D3-10*01 | 1 | 0.085 | ARNAIRIYGLLWFGELLYYGMDV |
| BG18-GL_Alt3_ | D3-10*02 | 3 | 0.044 | ARNAIRIYGITMFGELLYYGMDV |
| BG18-GL_Alt4_ | D3-10*02 | 3 | 11 | ARNAITMFGELLLGEYYYYGMDV |
| BG18-GL_Alt5_ | D3-10*01 | 2 | 1600 | ARNAIYYYGSGSYYEYYYYGMDV |

Red indicates D gene encoded residues. Blue indicates non-templated junction residues or SHM. Black indicates V_H_ or J_H_ encoded residues.

**Table S5. Commercial antibodies used in this study**.

| Antibody | Source | Identifier (cat# and RRID) |
| --- | --- | --- |
| mouse anti-human CD3 APC-Cy7 | BD Biosciences | cat# 557757  RRID:AB_396863 |
| mouse anti-human CD14 APC-Cy7 | BioLegend | cat# 301819  RRID:AB_493695 |
| mouse anti-human CD16 APC-eFluor780 | Thermo Fisher Scientific | cat# **47-0168-42**  RRID:AB_11220086 |
| mouse anti-human CD20 PerCP-Cy5.5 | BioLegend | cat# 302326  RRID:AB_893285 |
| mouse anti-human CD20 Alexa Fluor 488 | BioLegend | cat# 302316  RRID:AB_493227 |
| mouse anti-human CD27 PE-Cy7 | BioLegend | cat# 302837  RRID:AB_2561919 |
| goat anti-human IgD FITC | Southern Biotech | cat# 2030-02 |
| mouse anti-human IgG BV786 | BD Biosciences | cat# 564230  RRID:AB_2738684 |
| mouse anti-human IgG Alexa Fluor 700 | BD Biosciences | cat# 561296  RRID:AB_10612406 |
| mouse anti-human IgM BV605 | BD Biosciences | cat# 562977  RRID:AB_2737928 |
| mouse anti-human IgM PerCP-Cy5.5 | BD Biosciences | cat# 561285  RRID:AB_10611998 |
| Peroxidase AffiniPure Donkey Anti-Human IgG (H+L) | Jackson ImmunoResearch | cat# 709-035-149  RRID:AB_2340495 |
| mouse anti-human CD3 APC-Cy7 | BD Biosciences | cat# 557757  RRID:AB_396863 |
| mouse anti-human CD3 BV786 | BD Biosciences | cat# 563918  RRID:AB_2738487 |
| mouse anti-human CD4 BV711 | BioLegend | cat# 317439  RRID:AB_11219404 |
| Mouse anti-human CD4 APC-Cy7 | BioLegend | cat# 317418  RRID:AB_571946 |
| mouse anti-human CD8a APC-eFluor780 | Thermo Fisher Scientific | cat# **47-0088-42**  RRID:AB_1272046 |
| mouse anti-human CD8 APC-Cy7 | BD Biosciences | cat# 557760  RRID:AB_396865 |
| mouse anti-human CD38 APC | NHP Reagent resource | cat# PR-3801  RRID:AB_2819277 |
| mouse anti-human CD71 PE-CF594 | BD Biosciences | n/a |
| mouse anti-human PD-1 BV605 | BioLegend | cat# 329924  RRID:AB_2563212 |
| mouse anti-human CXCR5 PE-Cy7 | Thermo Fisher Scientific | cat# **25-9185-42**  RRID:AB_2573540 |
| Mouse anti-human IgM PE | BioLegend | cat# 314508  RRID:AB_493005 |
| Anti-rhesus IgG (H+L) | Bio-Rad | cat# AAI42  RRID:AB_322528 |
| AffiniPure F(ab’)_2_ Fragment Goat Antihuman IgG (H+L) | Jackson Immunoresearch | cat# 109-006-088  RRID:AB_2337549 |
| 6x-His tag monoclonal antibody | Invitrogen | cat# MA1-21315 |
| alkaline phosphatase (AP)-conjugated anti-Human IgG Fc fragment specific secondary | Jackson Immunoresearch | cat# 109-055-098  RRID:AB_2337608 |
| Anti-Human IgG (Fc) antibody | GE | cat# BR-1008-39 |
| His-tag Antibody | GenScript | cat# A00174  RRID:AB_914703 |
| TotalSeq-C0251 anti-human Hashtag 1 antibody | BioLegend | cat# 394661  RRID:AB_2801031 |
| TotalSeq-C0252 anti-human Hashtag 2 antibody | BioLegend | cat# 394663  RRID:AB_2801032 |
| TotalSeq-C0253 anti-human Hashtag 3 antibody | BioLegend | cat# 394665  RRID:AB_2801033 |
| TotalSeq-C0254 anti-human Hashtag 4 antibody | BioLegend | cat# 394667  RRID:AB_2801034 |
| TotalSeq-C0255 anti-human Hashtag 5 antibody | BioLegend | cat# 394669  RRID:AB_2801035 |
| TotalSeq-C0256 anti-human Hashtag 6 antibody | BioLegend | cat# 394671  RRID:AB_2820042 |
| TotalSeq-C0257 anti-human Hashtag 7 antibody | BioLegend | cat# 394673  RRID:AB_2820043 |
| TotalSeq-C0258 anti-human Hashtag 8 antibody | BioLegend | cat# 394675  RRID:AB_2820044 |
| TotalSeq-C0259 anti-human Hashtag 9 antibody | BioLegend | cat# 394677  RRID:AB_2820045 |
| TotalSeq-C0260 anti-human Hashtag 10 antibody | BioLegend | cat# 394679  RRID:AB_2820046 |
| TotalSeq-C0953 PE | BioLegend | cat# 405265 |
